# The potato sugar transporter SWEET1g affects apoplasmic sugar ratio and phloem-mobile tuber- and flower-inducing signals

**DOI:** 10.1093/plphys/kiae602

**Published:** 2024-11-07

**Authors:** Angelique Lauschke, Leonie Maibaum, Mira Engel, Luise Eisengräber, Sina Bayer, Aleksandra Hackel, Christina Kühn

**Affiliations:** Department of Plant Physiology, Institute of Biology, Humboldt-Universität zu Berlin, Philippstr. 13 Building 12, 10115 Berlin, Germany; Department of Plant Physiology, Institute of Biology, Humboldt-Universität zu Berlin, Philippstr. 13 Building 12, 10115 Berlin, Germany; Department of Plant Physiology, Institute of Biology, Humboldt-Universität zu Berlin, Philippstr. 13 Building 12, 10115 Berlin, Germany; Department of Plant Physiology, Institute of Biology, Humboldt-Universität zu Berlin, Philippstr. 13 Building 12, 10115 Berlin, Germany; Department of Plant Physiology, Institute of Biology, Humboldt-Universität zu Berlin, Philippstr. 13 Building 12, 10115 Berlin, Germany; Department of Plant Physiology, Institute of Biology, Humboldt-Universität zu Berlin, Philippstr. 13 Building 12, 10115 Berlin, Germany; Department of Plant Physiology, Institute of Biology, Humboldt-Universität zu Berlin, Philippstr. 13 Building 12, 10115 Berlin, Germany

## Abstract

The main phloem loader in potato, sucrose transporter StSUT1, is coexpressed with 2 members of the SWEET gene family: StSWEET11b, a clade III member of SWEET carriers assumed to be involved in sucrose efflux, and StSWEET1g, a clade I member involved in glucose efflux into the apoplast, that physically interacts with StSUT1. We investigated the functionality of SWEET carriers via uptake experiments with fluorescent glucose or sucrose analogs. Inhibition or overexpression of *StSWEET1g/SlSWEET1e* affected tuberization and flowering in transgenic potato plants. Isolation of the apoplasmic fluid by vacuum infiltration centrifugation revealed changes in the apoplasmic hexose composition and mono-to-disaccharide ratio, affecting sink strength. Downregulation of *StSWEET1g* expression affected the expression of SP6A, a tuberigen, and miR172 under long-day conditions, leading to early flowering and tuberization. A systematic screen for StSWEET1g-interacting protein partners revealed several proteins affecting cell wall integrity and strengthening. StSWEET1g and the main interaction partners were strongly downregulated during tuber development. We discuss whether StSWEET1g activity might be linked to cell wall remodeling during tuber development and the switch from apoplasmic to symplasmic phloem unloading.

## Teaser

Glucose efflux into the apoplast via a sugar transport protein affects phloem-mobile signaling molecules, flowering, and tuberization.

## Introduction

Sucrose is the main transport molecule for photoassimilates that are transported over long distances within plants via the phloem translocation stream. Sucrose transporters of the SUT gene family are described to be essential in the phloem loading process in source leaves of apoplasmic phloem loaders. A prerequisite for the proton-coupled phloem loading from the apoplasmic space into the conducting elements of the phloem via SUT proteins is the release of sucrose from cells where sucrose synthesis takes place, namely the mesophyll cells or alternatively from phloem parenchyma cells into the apoplast. This efflux is assumed to be catalyzed by SWEET carriers belonging to the phylogenetic clade III of SWEET efflux carriers (Chen et al. 2012). In Arabidopsis, AtSWEET11 and AtSWEET12 seem to be involved in sucrose export from phloem parenchyma cells allowing subsequent phloem loading in the sieve element–companion cell complex via the *Arabidopsis thaliana* sucrose transporter AtSUC2 (corresponding to SUT1 in other species). *Atsweet11/atsweet12* double knockout mutants are defective in phloem loading, and both sucrose efflux carriers are tightly coexpressed with the main phloem loader AtSUC2 (Chen et al. 2012).

According to phylogenetic analysis of a variety of plants, SWEET carriers were grouped in 4 clades sharing almost 27% to 80% in identity (Chen et al. 2010; Feng and Frommer 2015), and their assignment to one of the clades corresponds to the transported substrates: clade I and II mainly transport hexoses, whereas clade III carriers preferentially transport sucrose (Chen et al. 2010, 2012; Lin et al. 2014). Clade IV members apparently transport hexoses (mainly fructose) at the vacuolar membrane (Chen et al. 2010; Chardon et al. 2013; Guo et al. 2014). Apparently, the numerous members of the large gene family show only low functional redundancies.

Whether a similar division of labor with respect to efficient phloem loading or unloading is valuable in crop species as well is still unknown. More than 30 SWEET family members are described for potato and tomato plants (Manck-Götzenberger and Requena 2016; Ho et al. 2019). So far, only phloem unloading in sink organs of tomato plants has been investigated in detail: the SWEET carrier SlSWEET1a was shown to be highly expressed in young tomato leaves and in reproductive sink organs such as flowers. The carrier was localized at the plasma membrane (PM) of cells of the vascular tissue and is active in the low affinity transport of glucose (Ho et al. 2019). Downregulation of *SlSWEET1a* correlates with a reduction of hexoses in sink leaves and increased accumulation of source leaves, and it is assumed that SlSWEET1a makes part of the sugar unloading mechanisms in sink leaves of tomato (Ho et al. 2019). Both genomes, the potato and the tomato genomes, show a high degree of synteny (Manck-Götzenberger and Requena 2016). The synteny analysis indicated conserved gene arrangements between potato and tomato chromosomes, underscoring the genomic similarities within Solanaceae.

StSUT1 is not only involved in phloem loading but also a potential role in phloem unloading was suggested earlier (Kühn et al. 2003). The capacity of proton-coupled SUT1 transporter to mediate sucrose efflux was investigated in detail by electrophysiological *in vitro* measurements (Carpaneto et al. 2005). It is the question whether StSUT1-mediated sucrose phloem unloading might also be affected or supported by SWEET carriers.

It is known that the mechanism of phloem unloading in potato plants during tuber development switches from apoplasmic phloem unloading after tuber initiation to symplasmic phloem unloading later during tuber swelling (Viola et al. 2001). Flower and tuber induction in potato depends on the photoperiod and is triggered mainly by the phloem-mobile FT homolog SP6A (Navarro et al. 2011; Kloosterman et al. 2013). In a recent manuscript, the direct physical interaction between the tuberigenic SP6A protein with one of the SWEET carriers, StSWEET11b, was proven and this interaction is assumed to be important to prevent sucrose leakage into the apoplast and promotion of symplasmic unloading. Thereby, SP6A is involved in the source–sink communication affecting carbohydrate partitioning and it is known that the apoplasmic sugar content plays an important role in the determination of the sink demand (Abelenda et al. 2019).

Thus, elucidation of the involvement of members of the large SWEET gene family in phloem transport mechanisms in important crop plants such as potato plants is overdue. Here, we investigate the impact of glucose transporting SWEET1g/e as a member of clade I SWEET carriers on apoplasmic sugar ratio. Sucrose is known to affect the expression of miR172 (Garg et al. 2021) and SP6A (Abelenda et al. 2019). Therefore, changes in the apoplasmic sugar composition might directly affect the expression of phloem-mobile signaling molecules such as miR172, as well as SP6A leading to changes in tuberization and flowering behavior.

Elucidation of the SWEET1g/e interactome reveals a high amount of cell wall proteins, and the interaction between SWEET1g/e and these proteins often occurs at plasmodesmata. It is the question whether StSWEET1g is involved in the switch from apoplasmic to symplasmic phloem unloading during early stages of tuber development. A detailed expression analysis of StSWEET1g and its interaction partners during tuber swelling was performed. It is discussed to what extent changes in cellulose synthesis, pectin methylesterification, callose deposition, and other cell wall components can affect carbon partitioning and phloem loading as well as unloading strategies.

## Results

### Coexpression analysis and functionality

SWEET and SUT proteins form a complex coexpression network. Coexpression analyses using the TomExpress database (https://tomexpress.gbfwebtools.fr/) or the potato eFP Browser (https://bar.utoronto.ca/efp_potato/cgi-bin/efpWeb.cgi) revealed coexpression of StSUT1/SlSUT1, StSWEET1g/SlSWEET1e, and StSWEET11b/SlSWEET12a (Supplementary Figs. S1 and S2). Cloning of 3 members of the SWEET family that are coexpressed with the main phloem loading protein SUT1 in potato and tomato showed different substrate specificities. Whereas the member of the clade III of SWEET carriers, namely SlSWEET12a, is most likely involved in sucrose efflux into the apoplast, the clade I SWEET carriers, namely StSWEET1g and SlSWEET1e, are most likely engaged in hexose transport (Supplementary Fig. S3).

**Figure 1.**
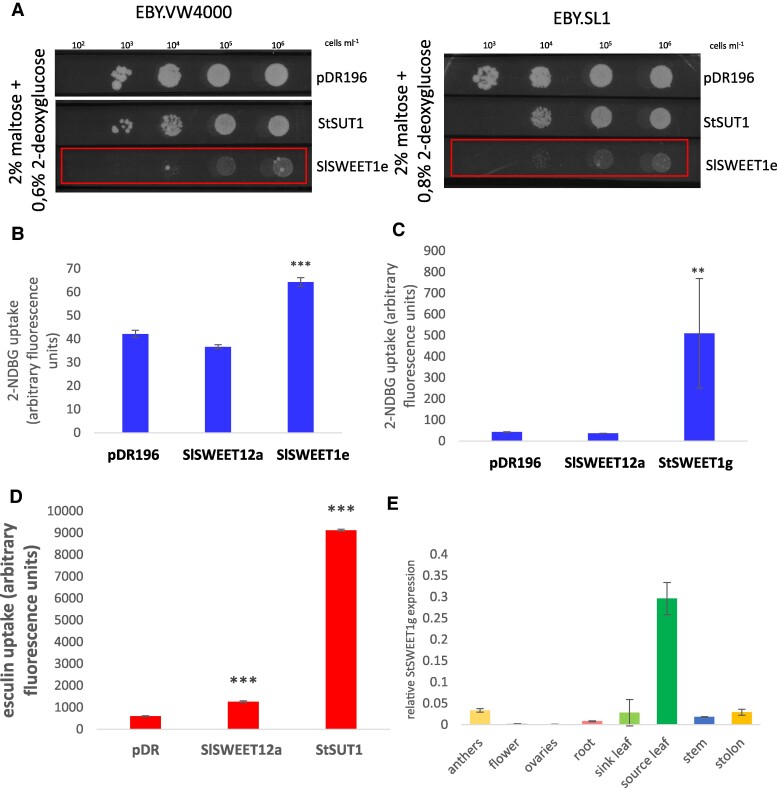
Functionality tests. **A)** Yeast complementation assays of sugar uptake defective yeast mutant strains EBY.SL1 (Krügel et al. 2013) and EBY.VW4000 (Wieczorke et al. 1999) revealed SlSWEET1e-mediated uptake of the toxic glucose analog 2-DG at various concentrations (rectangles), whereas the empty vector control pDR196, as well as the sucrose transporter StSUT1 in pDR196, is not able to take up the toxic compound allowing yeast growth even in the presence of 2-DG. **B)** Uptake of the fluorescent glucose analog 2-NDBG in yeast strain EBY.VW4000 measured at 5.5 mm substrate concentration at pH 7 reveals higher uptake by SlSWEET1e than by the empty vector and SlSWEET12a. **C)** Uptake of the fluorescent glucose analog 2-NDBG in yeast strain EBY.VW4000 via StSWEET1g is higher than the one of the empty vector and SlSWEET12a. **D)** Esculin uptake in yeast cells (strain EBY.SL1) with 1 mm of esculin at pH 5 by SlSWEET12a suggests sucrose transport capacity. StSUT1 was used as a positive control. StDev of 5 replicates is given. Experiments were reproduced twice. **E)** Tissue-specific expression of StSWEET1g in potato plants as determined by qPCR. StDev is given. *t* test with **P < 0.01, and ***P < 0.001.

In order to test its ability to complement sucrose uptake defective yeast mutants, we used the hexose transporter deficient (hxt0) strain EBY.VW4000 (Wieczorke et al. 1999; Boles and Oreb 2018), and the mutant strain EBY.SL1 defective in glucose transport, maltose transport, and invertase activity (Krügel et al. 2012) for complementation assays (Fig. 1) offering various carbon containing compounds at increasing concentrations. Whereas the transformed yeast cells are growing well in the presence of maltose (2%) even if transformed with the empty vector, no growth is observed if glucose or sucrose is supplied as the sole carbon source (Supplementary Fig. S4A). By increasing the amount of sugar substrate (2%, 4%, 6%, 8%, and 10%), we hoped to identify a low affinity transport component. SlSWEET1e was not able to mediate sugar uptake and yeast growth to the 2 strains, and EBY.VW400 or EBY.SL1, even at high sugar concentrations, was not able to complement either of the 2 strains.

Addition of the toxic glucose analog 2-deoxyglucose (2-DG), however, reduced yeast viability substantially, if the yeast cells were transformed with SlSWEET1e (Fig. 1A) or StSWEET1g (Supplementary Fig. S4B), but not in the presence of the empty vector alone or the sucrose transporter StSUT1 in pDR196 (Fig. 1A), suggesting that SlSWEET1e and StSWEET1g are glucose transporters. Thereby, previous investigations with tomato SWEET1 carriers were successfully confirmed (Ho et al. 2019) and comparable results provided for StSWEET1g.

Yeast uptake experiments with fluorescent sucrose or glucose analogs confirmed this assumption (Fig. 1, B to D). Yeast uptake experiments have been performed using fluorescent sucrose or glucose analogs, esculin or 2-NDBG, respectively. Thereby, it could be shown that SlSWEET12a is able to take up esculin at a concentration of 3 mm (Fig. 1C), whereas SlSWEET1e from tomato and its putative ortholog from potato, StSWEET1g (Manck-Götzenberger and Requena 2016), are able to take up the fluorescent glucose analog 2-NBDG (Fig. 1, B and C).

Reverse transcription quantitative PCR (RT-qPCR) analysis of tissue-specific *StSWEET1g* expression confirmed the tight coexpression with *StSUT1* with the highest expression level in source leaves (Fig. 1E). Since C-terminal fusions of fluorescent proteins reflect more reliably the subcellular localization of native proteins than N-terminal tagging (Palmer and Freeman 2004), the subcellular localization of StSWEET1g, SlSWEET1e, and their interacting proteins was performed with C-terminal fusions to YFP.

### Phenotype of transgenic plants with altered *StSWEET1g* expression

In order to elucidate the role of the monosaccharide transporter of the clade I SWEET family tightly coexpressed with the main phloem loading SUT1, transgenic potato plants carrying RNAi or overexpression constructs were generated and analyzed. The rich genetic similarity between potato and tomato SWEETs, with identities reaching up to 97%, unlocks the potential for using orthologs from both plants in research. For instance, the functional equivalence between SlSWEET1e in tomato and its ortholog StSWEET1g in potato was explored, allowing us to use *SlSWEET1e* as RNAi constructs in transgenic potato plants (Supplementary Fig. S5A). Sequence identity between the 2 genes is 95.9% at the nucleotide level (and 97.2% identity for the fragment used for RNAi) and 97% at the amino acid level. In order to prevent cosuppression events, overexpression experiments were conducted with the tomato orthologous clone *SlSWEET1e* (Manck-Götzenberger and Requena 2016) expressed under control of the CaMV35S promoter and translationally fused to the YFP protein in the binary pK7YWG2.0 vector (Karimi et al. 2005) used for stable plant transformation. Downregulation of the endogenous *StSWEET1g* gene was tested by qPCR using *StSWEET1g*-specific primers, and overexpression of *SlSWEET1e* was quantified using *SlSWEET1e*-specific primers in qPCR experiments. Although sequence similarities between SWEET carriers are generally high, downregulation of *StSWEET1g* was highly specific (Supplementary Fig. S5, B and C).

**Figure 2.**
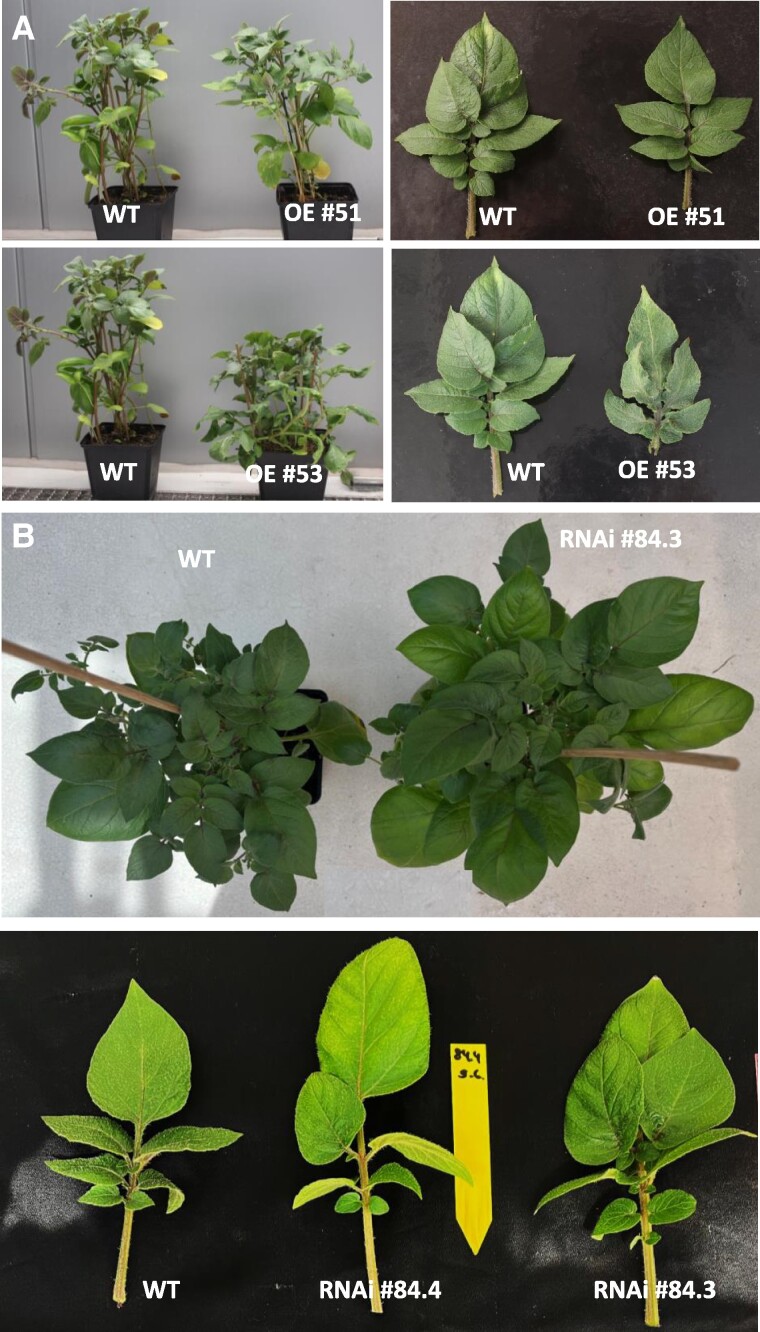
Phenotype of transgenic potato plants. Phenotype of SWEET1e-overexpressing **A)** and SWEET1g-RNAi plants **B)** grown for 6 wk under LD conditions in the green house. Source leaves of SWEET1e overexpressors are slightly smaller than wild-type leaves, whereas source leaves of SWEET1g-RNAi plants are slightly broader. Changes of leaf morphology are not significant.

The phenotypic changes were observed after 6 wk of growth under long-day (LD) conditions in the green house (Fig. 2). The leaf morphology and plant height were only slightly affected, and changes were only marginal (Fig. 2; Supplementary Fig. S6). Since the potato variety Désirée belongs to the *Solanum tuberosum* subspecies *tuberosum*, tubers can be produced even under noninductive LD conditions. This subspecies is not under strict photoperiodic control.

**Figure 3.**
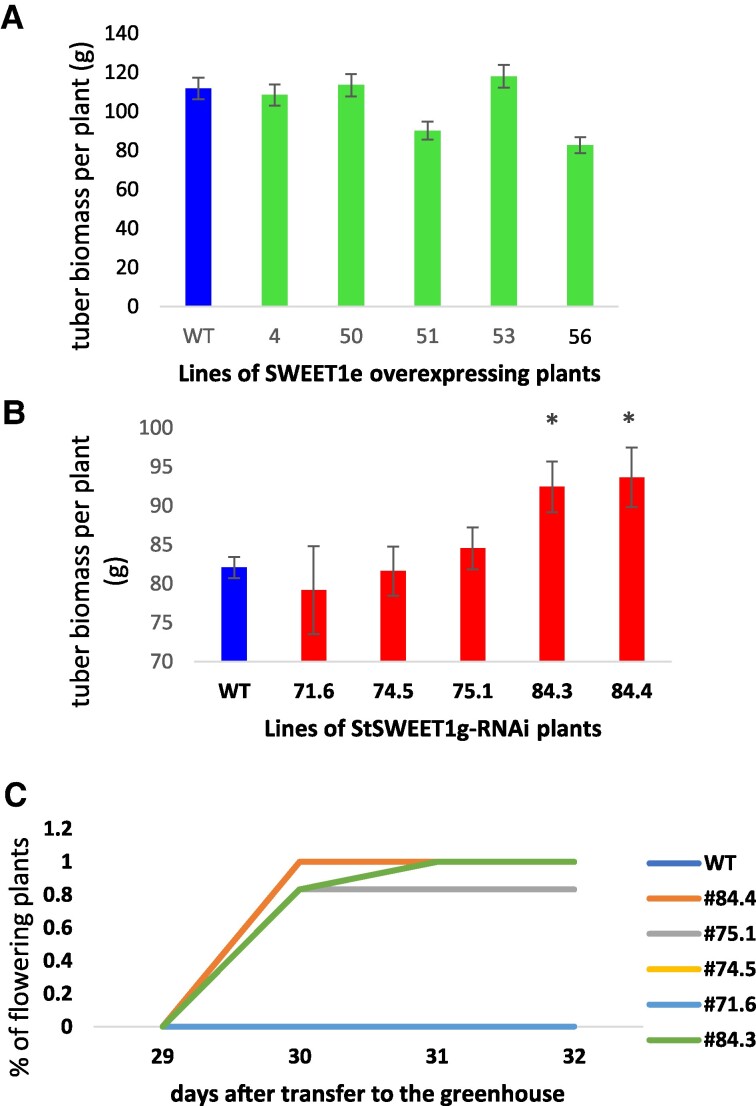
Tuberization and flowering behavior. Tuber biomass and flowering behavior of transgenic plants grown under LD conditions. **A)** Tuber biomass of *SlSWEET1e*-overexpressing plants is slightly reduced in 2 out of 5 transgenic lines. Changes were not significant. **B)** Tuber biomass of *StSWEET1g*-RNAi plants is significantly increased in 2 out of 5 transgenic lines (*t* test with *P < 0.05). SEM is given in **A)** and **B)**. **C)** Flowering behavior of *StSWEET1g*-RNAi plants under LD conditions. Three out of 5 transgenic lines of the transgenic plants were early flowering with less numbers of leaves produced at flowering time. Wild-type plants were not flowering under these conditions. Experiments were repeated at least 3 times independently with similar results and with 6 plants per line. One representative experiment is given.

The tuber yield of 4 independent *StSWEET1g*-RNAi lines were increased compared to WT plants under LD conditions (Supplementary Fig. S6), and these observations were reproduced in several additional experiments, where 2 out of 5 lines of *StSWEET1g*-RNAi plants show significant higher tuber biomass (Fig. 3B), whereas the *SlSWEET1e*-overexpressing plants tend to produce a decreased total tuber biomass (Fig. 3A; Supplementary Fig. S6B). The number of tubers produced was significantly reduced in both sets of plants (Supplementary Fig. S6C). Tuber yield was inversely correlated with the expression level of SlSWEET1e/StSWEET1g. Whereas Désirée WT plants and *SlSWEET1e*-overexpressing plants did not flower under LD conditions, 3 out of 5 transgenic *StSWEET1g*-RNAi plants flowered early (Fig. 3C; Supplementary Fig. S7).

Growth experiments were conducted under short-day (SD) conditions as well. Here, the general plant and leaf phenotype showed similar results as under LDs (Supplementary Fig. S8). It was obvious that *StSWEET1g*-RNAi plants were early senescent, but they were not flowering and tuber biomass was not significantly increased as under LDs. Early senescence and accelerated development could be the reason for the early flowering phenotype under LD conditions. This was tested under LD conditions, but the number of leaves at flowering was not affected. Leaves of S*tSWEET1g*-RNAi plants were slightly broader than WT leaves (Supplementary Fig. S8B), whereas the leaves of *SlSWEET1e*-overexpressing potatoes seem to be slightly narrower (Supplementary Fig. S8B).

Under SD conditions, the tuber yield of *SlSWEET1e*-overexpressing plants was slightly smaller than the one of WT plants, and they produced aerial stolons and tubers (Supplementary Fig. S8, C and D), a phenomenon that was already described for potato plants overexpressing miR156 under the CaMV35S promoter (Eviatar-Ribak et al. 2013).

### Determination of the content of soluble sugars and starch

The total amount of sugars of source leaves of *SlSWEET1e*-overexpressing as well as *StSWEET1g*-RNAi plants was measured enzymatically (Stitt et al. 1989) regardless of compartmentalization (Supplementary Fig. S9). Whereas the amount of hexoses tends to be increased in *SlSWEET1e*-overexpressing plants (Supplementary Fig. S9A), it apparently decreased in *SWEET1g*-RNAi plants (Supplementary Fig. S9B). The fact that the amount of sucrose remained more or less unaffected in both sets of plants is an additional argument for SWEET1e/g being mainly involved in hexose transport (Supplementary Fig. S9).

### Isolation of the apoplasmic fluid by vacuum infiltration centrifugation

Plasma membrane-specific SWEET carriers are assumed to be mainly involved in the efflux of sugars into the apoplasmic space (Chen et al. 2012). Therefore, the apoplasmic sugar content should be quantified in order to dissect out the StSWEET1g function more precisely.

**Figure 4.**
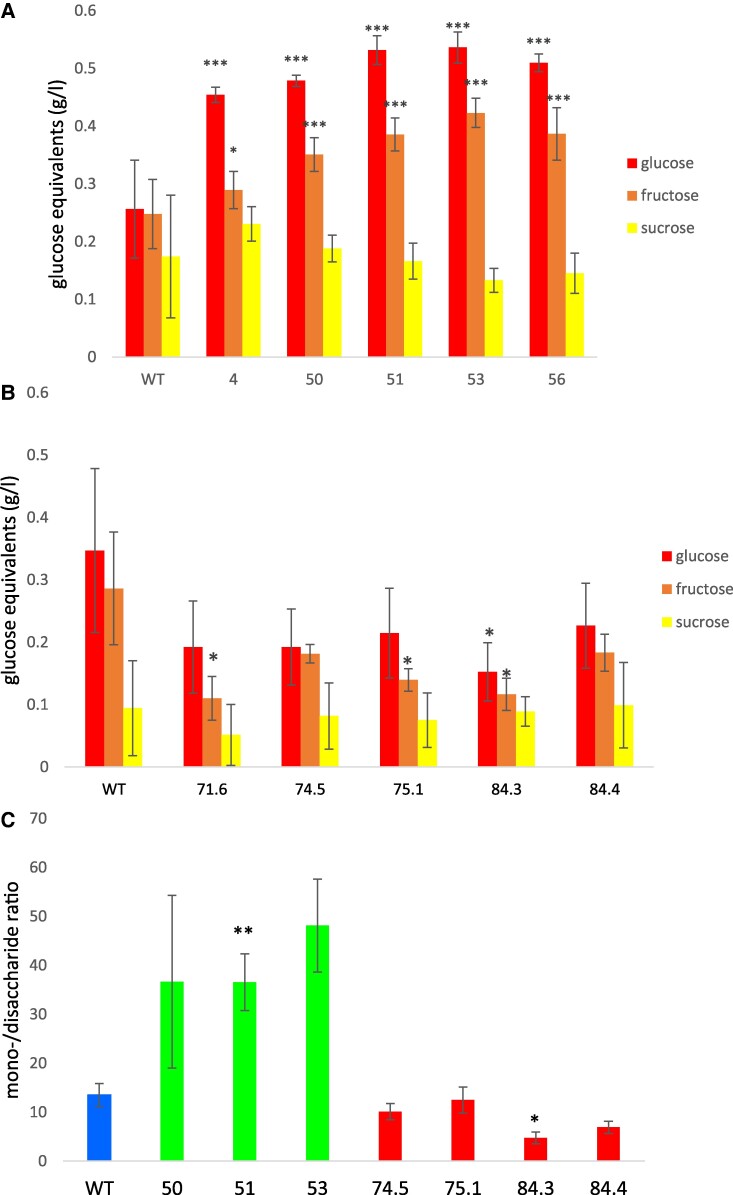
Sugar concentration in the apoplasmic fluid. **A)** Apoplasmic sugar content of SlSWEEET1e-overexpressing plants grown under LD conditions as determined by vacuum infiltration centrifugation. Only monosaccharides were significantly increased, whereas sucrose levels are almost unaffected. **B)** Apoplasmic sugar content of StSWEET1g-RNAi plants under SDs. Only monosaccharides were significantly decreased in StSWEET1g-RNAi plants. **C)** Mono-to-disaccharide ratio of apoplasmic sugars of Désirée wild-type, SlSWEET1e-overexpressing (left), and StSWEET1g-RNAi plants (right) grown under SD conditions. *t* test with *P < 0.05, **P < 0.01, and ***P < 0.001. StDev is given, and 8 biological replicates have been measured.

We established the technique for isolation of the apoplasmic fluid according to Abelenda et al. (2019) using aerial parts of the transgenic plants. Indeed, only hexoses and thereby the mono-to-disaccharide ratio were significantly affected in those plants (Fig. 4) with *SlSWEET1e*-overexpressing plants with more hexoses in the nongreen apoplasmic fluid (Fig. 4A) and *StSWEET1g*-RNAi plants with reduced hexose content and reduced mono-to-disaccharide ratio (Fig. 4, B and C). Similar results were obtained when selected lines of the transgenic plants were grown under LD conditions and apoplasmic fluid was isolated (Supplementary Fig. S9C). Thus, apoplasmic mono-to-disaccharide ratio seems to be crucial for the induction of flowering and tuberization.

### *SP6A* expression is affected under LD conditions

**Figure 5.**
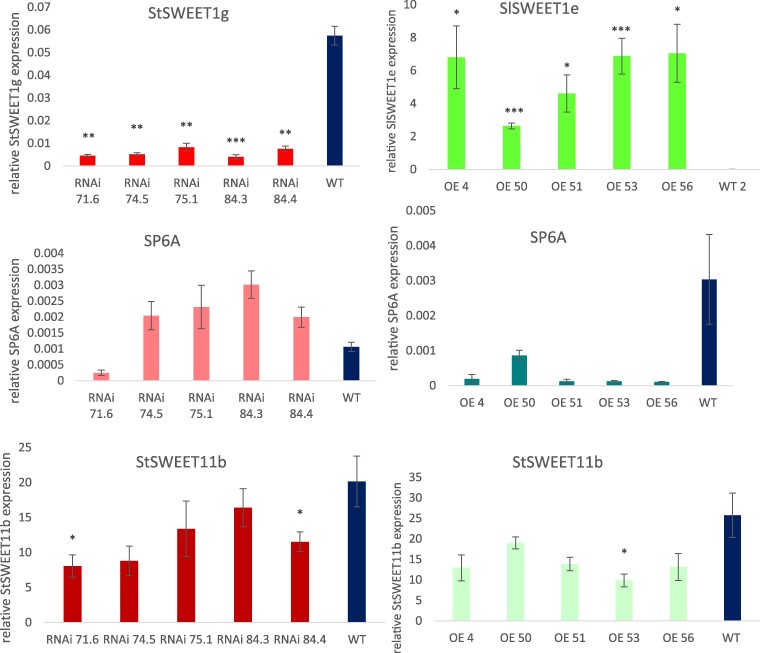
Transcript quantification via qPCR. Real-time qPCR analysis using RNA from source leaves from transgenic plants grown under LD conditions. Quantification of SlSWEET1e, StSWEET11b, and StSP6A transcripts in SlSWEET1e-overexpressing plants (right panels). Quantification of StSWEET1g, StSWEET11b, and StSP6A in StSWEET1g-RNAi plants (left panels). The average of 6 replicates is shown. TEF was used as a reference gene, and SEM is given. *t* test with *P < 0.05, **P < 0.01, and ***P < 0.001.

Whereas the tuber-inducing FT homolog *SP6A* is not expressed under LD conditions in the strictly photoperiod-dependent subspecies *andigena* (Navarro et al. 2011), *SP6A* expression is well documented under LD conditions in the variety Désirée (*S. tuberosum* ssp. *tuberosum*). *SP6A* is a phloem-mobile signaling molecule that is preferentially under SD conditions transported toward belowground stolons to induce tuber production (Navarro et al. 2011). Quantification of this tuberigenic molecule via qPCR analysis revealed reduced transcript levels in *SlSWEET1e*-overexpressing lines (Fig. 5), whereas transcript levels are increased in *StSWEET1g*-RNAi lines (Fig. 5).

The qPCR analysis was repeated under SD conditions with less significant changes in *SP6A* expression compared to WT plants (Supplementary Fig. S10). It is interesting to note that the transcript level of the potato *StStSWEET11b*, which is postulated to directly interact with SP6A (Abelenda et al. 2019), is downregulated in *StSWEET1g*-RNAi plants grown under SD conditions (Supplementary Fig. S10), suggesting a coregulation of *StSWEET1g* and *StSWEET11b*, which acts as sucrose efflux carrier, under these conditions.

**Figure 6.**
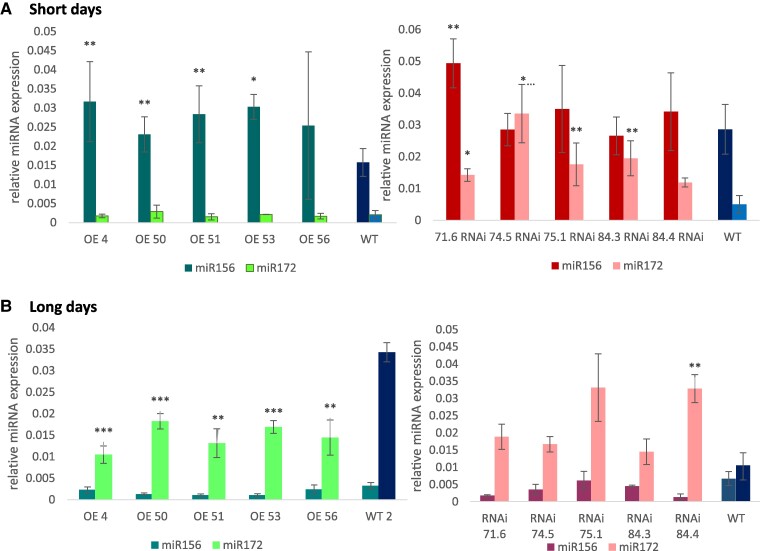
Quantification of mature miRNAs. Real-time qPCR analysis of phloem-mobile microRNAs in source leaves of *SlSWEET1e*-overexpressing (left panels ) and *StSWEET1g*-RNAi (right panels) plants grown under SD **A)** or LD **B)** conditions. Note that miR156 is increased in SlSWEET1e overexpressors under SD conditions **A)**, whereas miR172 is increased in StSWEET1g-RNAi plants regardless of the photoperiod **A** and **B)**. Increased levels of miR172 correlate with increased tuber yield of *StSWEET1g*-RNAi plants (Fig. 3B; Supplementary Fig. S6). 5SrRNA was used as a reference gene. Average of 6 replicates is shown, and StDev is given. *t* test with *P < 0.05, **P < 0.01, and ***P < 0.001.

Attenuated changes in *SP6A* expression under SDs correlate with attenuated effects on tuber production in both sets of plants. It is remarkable that *SlSWEET1e*-overexpressing plants develop aerial stolons and tubers if grown under SD conditions (Supplementary Fig. S8, C and D). This was earlier correlated with overexpression of miR156 (Eviatar-Ribak et al. 2013), and quantification of the mature miR156 in *SlSWEET1e*-overexpressing plants indeed revealed overaccumulation of *miR156* under SD conditions (Fig. 6A). In contrast, in *StSWEET1g*-RNAi plants, the amount of *miR172* is higher than in WT plants both under SD and LD conditions (Fig. 6B). Both miRNAs are described to act sequentially (Wu et al. 2009).

### Most of the SWEET1e-interacting proteins are cell wall resident or PM associated

SWEET carriers are known to form functional trimeric complexes (Tao et al. 2015). In case of the vacuolar glucose transporter OsSWEET2b from rice, the structural and biochemical analyses showed homomeric trimers to be functional (Tao et al. 2015).

**Figure 7.**
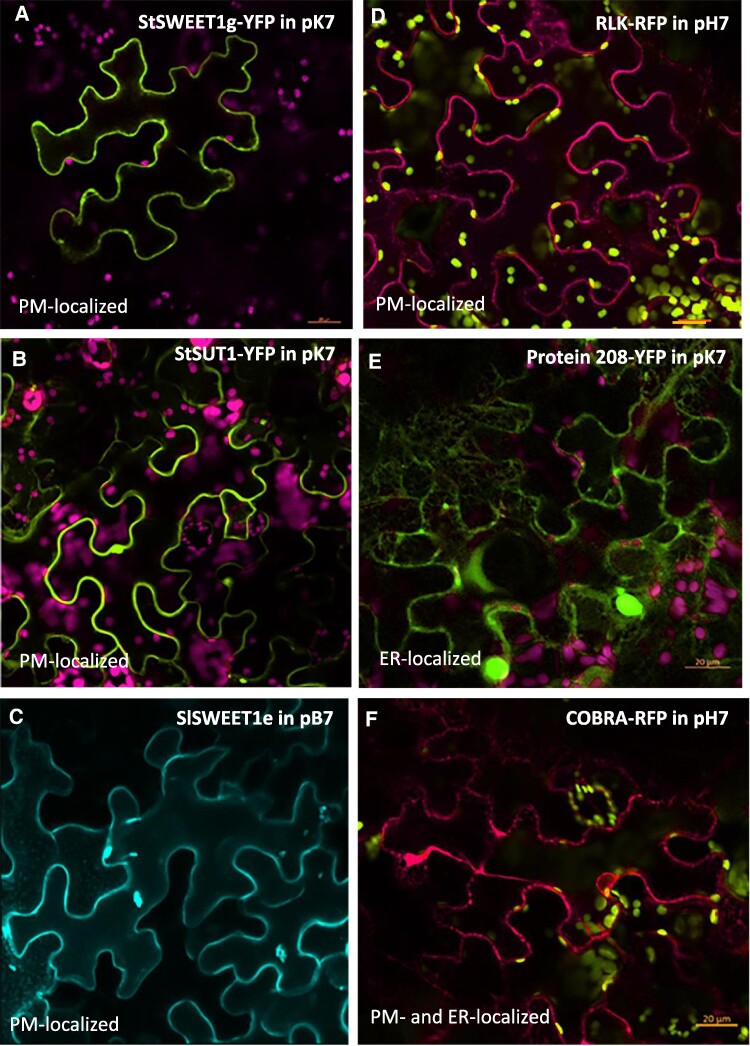
Subcellular localization of sugar carriers and their putative interacting proteins by confocal microscopy using fluorescent fusion constructs. The RLK **D)** and the COBRA protein **F)** were fused to RFP in the binary vector pH7, whereas the StSWEET1g **A)**, StSUT1 **B)**, and protein 208 **E)** were fused to YFP using the binary vector pK7. SlSWEET1 was fused to CFP **C)**. **D** and **F)** Chlorophyll is shown in yellow, and RFP fluorescence in red. **A**, **B**, and **E)** YFP fluorescence is shown in green, and chlorophyll autofluorescence is shown in purple. Confocal pictures were taken 3 d after infiltration. Note that RLK localization is restricted to the plasma membrane, whereas protein 208 and COBRA are also detectable in the ER. Scale bar corresponds to 20 *µ*m.

Subcellular localization of StSWEET1g and SlSWEET1e revealed plasma membrane-specific localization of the monomeric protein (Fig. 7, A and C). In order to test whether SWEET1g/e can form homomeric complexes as well, bimolecular fluorescence complementation (BiFC) assays were performed using VYNE and VYCE vectors described earlier (Gehl et al. 2009). StSWEET1g/SlSWEET1e, which is usually found at the plasma membrane (Fig. 7, A and C), forms homodimeric complexes preferentially found in intracellular structures (Supplementary Fig. S11A). As previously shown for plant sucrose transporters SUT1 and SUT4 (Garg et al. 2020), the homodimer formation of SlSWEET1e obviously promotes internalization of the complex and separation from the plasma membrane.

**Figure 8.**
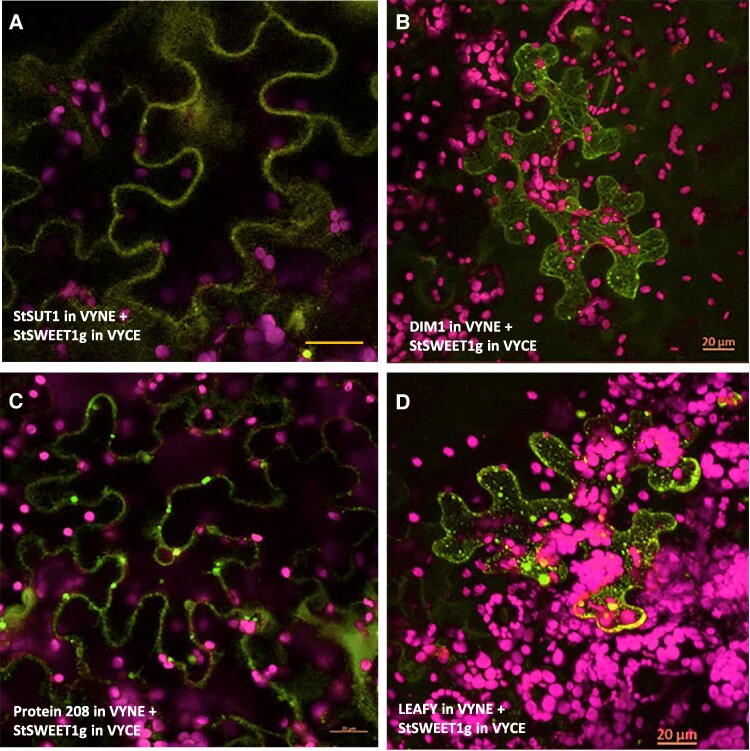
Protein–protein interactions of StSWEET1g in planta by BiFC. **A)** Heteromer formation between StSWEET1g interacting with StSUT1 at the plasma membrane. **B)** Confirmation of interaction between DIM1 and StSWEET1g. **C)** Confirmation of interaction between the protein 208 and StSWEET1g. **D)** Confirmation of interaction between StSWEET1g and LEAFY. Pictures were taken 3 to 4 d after infiltration. Note that interactions mainly take place in the cell periphery and that interaction changes subcellular localization of interaction partners (compared to Fig. 7). YFP fluorescence from BiFC experiments is shown in yellow, and chlorophyll autofluorescence is shown in purple. Note that interactions with all candidates were also confirmed for SlSWEET1e (Supplementary Figure S11). Scale bar corresponds to 20 *µ*m.

The main phloem loader StSUT1 that is coexpressed with StSWEET1g was also tested as direct interacting partner, although it is not to be expected that both proteins share the same cellular compartment. Again, fluorescence complementation was clearly confirmed and interaction takes place at the plasma membrane (Fig. 8; Supplementary Fig. S11B).

In order to learn more about the physiological function of StSWEET1g/SlSWEET1e, a systematic split ubiquitin screen for interacting proteins was performed using a potato cDNA library generated from all potato tissues having a complexity of 6 × 10^5^ and an average insert size of 1.3 kb (Krügel et al. 2012). The number of primary transformants was 6.24 × 10^5^ colonies. The screen was performed without 3-aminotriazol (3-AT), and 200 yeast colonies were selected and tested for the ability to grow on 10 mm 3-AT preventing autoactivation of the *his3* gene. Restriction analysis of the cDNA inserts was performed in *Escherichia coli*, and identical inserts were grouped together and analyzed by sequencing. Yeast strain L40ccu ura^−^ was retransformed as described earlier (Krügel et al. 2012). Finally, 29 different cDNA inserts could be confirmed after retransformation and sequencing, which are envisaged to be confirmed alternatively (Table 1). Among the 29 candidates, 7 are predicted to be confined to the plasma membrane and 7 others predicted to be cell wall-associated proteins.

**Table 1.** StSWEET1g/SlSWEET1e-interacting proteins identified using the split ubiquitin system (only cDNAs inserted in the same reading frame as the ubiquitin sequence were selected)

Number	Number of independent colonies	Accession number	Name (putative function)	Compartment (predicted)	Confirmation (BiFC)
1	9	NM_001288057 PGSC0003DTM400036749	LEAFY/FLORICAULA	SAM	Yes, at PD
2	6	SoltuDM03G015630.1 PGSC0003DTM400028031 XM_006362054.2	PMEI	Cell wall	
3	4	XM_006366928.2	Anti-Müllerian hormone receptor type 2 3 TMDs	PM	
4	2	Solyc08g066840.2.1 Solyc08g066840.2.1	TIP4-1 aquaporin	Vacuole	
5	2	PGSC0003DTM400007185 NM_001288541.1	StPDI1	ER PM	Yes
6	2	XM_006361128.2 PGSC0003DMT400005398	Glycine-rich cell wall structural protein	Cell wall	
7	2	PGSC0003DTM400012730 NM_001318651.2	AAP1	PM	
8	2	PGSC0003DTM400011358 XM_006366880.2	Selenoprotein T		
9	1	XM_004250283.4	Selenoprotein K, 1 TMD		
10	1	XM_006339552.2	Receptor-like kinase 2 TMDs	PM	Yes, at PD
11	1	XM_006343517.2	Transmembrane protein 208, 4 TMDs	Cell wall, this work: PM, PD	Yes, at PD
12	1	XM_006363519.2	COBRA Protein COBRA-like 7 1-2 TMDs, GPI-anchored	Cell wall, this work: ER/PM	Yes
13	1	XM_006361662.2	Cellulose synthase A-like catalytic subunit (UDP-forming)	Cell wall	
14	1	XM_015314363.1	G3BP-like protein, soluble, RNP granule endoribonuclease	Cell wall	
15	1	XM_015225605.2 LOC107024615	Defensin-like protein *Solanum pennelli*	Cell wall	
16	1	XM_006343909.1	PIP-type pTOM75 PIP1-5, 6 TMDs	PM	
17	1	XM_006338475.2	WAT1-related Umami-type transporter Drug/metabolite transporter	PM	
18	1	XM_006350438.2	Molybdate anion transporter like	PM	
19	1	PGSC0003DTM400052223 NM_001288206.1	Mitochondrial import receptor subunit TOM20-like	Mitochondria	
20	1	XM_006358751.2	Glucan water dikinase 3	Plastid	
21	1	XM_015314115	CORA protein		
22	1	PGSC0003DMT400015075	PSI subunit O	Plastid	
23	1	XM_006350751.2 PGSC0003DTM400078206	Tubulin beta-2-chain like		
24	1	CP055242.1	Adenosylhomocysteinase		
25	1	XM_006352030.2	Probable plastid lipid-associated protein	Plastid	
26	1	KAH0723560	Hypothetical protein		
27	1	JX683432.1	Light-induced PS II StDT110	Plastid	
28	1	XM_006340096.2	E3 ubiquitin ligase-like unknown protein		
29	1	XM_015314355.1	TCP7-like transcription factor, soluble	Nucleus	

Sorting according to the frequency of appearance in independent yeast colonies. Result of 50 colonies in total.

### Subcellular localization of interaction partners

Table 1 shows all StSWEET1g/SlSWEET1e-interacting proteins identified by the Split ubiquitin system arranged according to the frequency of appearance in independent yeast colonies. Eight out of 29 candidates were identified several times independently. The LEAFY transcription factor was identified as the prevalent interacting protein, while a cell wall pectin methylesterase inhibitor (PMEI) was the second most abundant interaction partner. In total, 26% to 28% of the interacting proteins are predicted to be cell wall associated, and some of the still unknown interaction partners were selected for further subcellular analysis.

The unknown receptor-like kinase (RLK) (XM_006339552.2) is predicted to have 2 transmembrane-spanning domains, to be able to bind to ATP and to ß-glucan molecules according to in silico analyses. Fusion to RFP helped to localize the RLK subcellularly (Fig. 7D) showing targeting exclusively to the plasma membrane.

Another interacting protein, the unknown transmembrane protein 208 (XM_006343517.2), is predicted to have 4 transmembrane-spanning domains and an assumed function in cellulose biosynthesis and/or autophagy. A translational YFP fusion construct is targeted to the plasma membrane and the endoplasmic reticulum (ER) (Fig. 7E).

Finally, the glycosylphosphatidylinositol (GPI)-anchored COBRA protein (XM_006363519.2) predicted to have 1 to 2 TMDs is assumed to play a role in cellulose synthesis and carbon partitioning (Julius et al. 2021) was fused to RFP and localized at the plasma membrane and the ER (Fig. 7F).

### Confirmation of interaction by BiFC experiments

Five out of 29 interaction partners have been confirmed by in planta experiments using BiFC. Intriguing to see that the subcellular localization of interaction partners changes depending on association to StSWEET1g (Fig. 8). Most of the candidates were also able to interact with SlSWEET1e, the ortholog from tomato (Supplementary Figs. S11 and S12).

The still unknown RLK (XM_006339552.2), which is assumed to be callose binding and which in localization experiments is strictly confined to the plasma membrane (Fig. 7D), was confirmed to be able to interact with SlSWEET1e in the cell periphery. The distribution over the plasma membrane appears here uneven in a spotty pattern (Supplementary Fig. S11D), and the ER membranes are also labeled.

Also the interaction between SlSWEET1e and the COBRA protein shows an uneven distribution at the plasma membrane (Supplementary Fig. S11F). Interaction with the LEAFY transcription factor, although unexpected, was confirmed, but interaction is not detectable in the nucleus as expected for a transcription factor, but in large aggregates and in the cell periphery in a punctate manner (Fig. 8D; Supplementary Fig. S11E).

Regarding the interaction experiments with protein 208 (XM_006343517.2) and DIM1, a plasmodesmal localization is assumed (Fig. 8; Supplementary Fig. S11, C and G). In order to test this hypothesis, aniline blue staining of plasmodesmal callose was performed and colocalization experiments confirmed plasmodesmal colocalization of the heteromeric complex between protein 208 and the SlSWEET1e protein in both orientations (Supplementary Fig. S11, H and I).

### Expression of StSWEET1g interactors during tuber development

Several SWEET carriers are among the deregulated genes (DEGs) during tuber development as recently determined by RNA-seq experiments (Shirani-Bidabadi et al. 2024): StSWEET1g is downregulated during 3 developmental stages of tuber development (Shirani-Bidabadi et al. 2024) (Supplementary Figs. S13 and S14). The list of DEGs deregulated during developmental stages I to III also contains several of the SWEET1g-interacting proteins: cellulose synthase A, WAT1-related protein, COBRA protein, glycine-rich structural cell wall protein, and many others (Shirani-Bidabadi et al. 2024).

Several of the StSWEET1g-interacting proteins are postulated to be involved in cellulose biosynthesis (cellulose synthase A, COBRA, and protein 208), pectin methyl esterification (PMEI), secondary cell wall formation (WAT1), or cell wall components (arabinogalactan AGP16) and therefore assumed to strengthen the cell wall stiffness (Supplementary Fig. S15). It is the question whether those interacting partners are also coexpressed with StSWEET1g.

In order to get insights in their role during tuber development, a detailed expression analysis was performed during 8 different developmental stages classified according to Kloosterman et al. (2005) (Supplementary Fig. S13). The switch from apoplasmic to symplasmic phloem unloading in potato is described to occur between developmental stages II and IV (Viola et al. 2001).

The qPCR expression analysis revealed that not only the transporters for sucrose (StSUT1 and StSUT4) and glucose (StSWEET1g) but also the StSWEET1g-interacting partners WAT1, RLK, and COBRA are strongly downregulated during tuber development (Supplementary Fig. S14), suggesting that strengthening of cell walls is diminished during tuber swelling.

## Discussion

### Function of StSWEET1g

Although SWEET carriers belong to large gene families (Chen et al. 2010; Feng and Frommer 2015), it is interesting to see a concise phenotype after manipulation of only one single member of the clade I of putative glucose transporting SWEET carriers.

In potato plants, the role of StSWEET11 has been well investigated (Abelenda et al. 2019). StSWEET11 is a sucrose transporter belonging to clade III, whereas StSWEET1g belongs to clade I and is postulated to be a glucose transporter (Eom et al. 2015; Manck-Götzenberger and Requena 2016).

The phenomenon of tuberization in potato is regulated in a photoperiod-dependent manner in a similar way as it is described for the induction of flowering. Induction of tuberization depends on the photoperiod, the availability of assimilates, and the phloem-mobile FT homolog SP6A (Navarro et al. 2011). During tuber development, the mode of phloem unloading switches from apoplasmic in first stages to symplasmic phloem unloading at later developmental stages (Viola et al. 2001). A recent manuscript describes the direct physical interaction between the tuberigenic SP6A protein with the sucrose transporter StSWEET11 to be involved in this switch (Abelenda et al. 2019). Binding of SP6A to StSWEET11 apparently blocks sucrose leakage out of the phloem and thereby promotes symplasmic unloading of sucrose in developing tubers (Abelenda et al. 2019).

It has been stated that sucrose is required for *SP6A* expression and transport blockage of StSWEET11 RNAi plants lead to a lowered amount of sucrose in companion cells (CCs) and repressed SP6A expression (Abelenda et al. 2019). Most likely, induction of the phloem-mobile SP6A by sucrose occurs via the miR172-AP2 module in source leaves, since accumulation of mature miR172 is strongly sucrose dependent in potato (Garg et al. 2021).

The role of StSWEET1g in this context could be the regulation of the sink strength by modulation of the apoplasmic glucose content (Supplementary Fig. S16). It is known that the main phloem loader from Arabidopsis, AtSUC2, is repressed by high glucose levels in the mesophyll (Wingenter et al. 2010). It has been hypothesized that high glucose levels are a result of sucrose cleavage in the apoplast, which initiates a signal cascade resulting in the inhibition of AtSUC2 and downregulation of photosynthesis. Therefore, glucose accumulation in the apoplast represents a signal for low sink demand and/or disrupted assimilate allocation due to external stimuli (Fernie et al. 2020). This would explain the effect of glucose transporter StSWEET1g on StSUT1 activity and perhaps also explains the direct physical interaction with partners involved in stress tolerance, like StPDI1 (Eggert et al. 2016), and mobilization of stored carbohydrates (via the glucan water dikinase).

Higher expression of *StSWEET1g* will result in higher apoplasmic glucose concentrations, thereby signaling low sink demand resulting in *SP6A* repression (Supplementary Fig. S16). Vice versa, in *StSWEET1g*-RNAi plants, the apoplasmic glucose content is lowered, which exhibited higher *SP6A* expression and thereby tuberization. This is exactly what was observed when analyzing the transgenic plants.

The early senescence observed in all *StSWEET1g*-RNAi lines under SD conditions suggests a potential acceleration of natural ageing processes in these plants. Notably, *SlSWEET1e*-overexpressing lines 50 and 51 exhibited a unique phenomenon of producing aerial tubers, a trait typically associated with the overexpression of the age-dependent miR156 (Eviatar-Ribak et al. 2013). This microRNA is known to regulate juvenility in plants and plays a crucial role in the transition from vegetative to reproductive phases in plant development and seems to be sugar dependent (Yang et al. 2013).

The observation of aerial tuber formation in *SlSWEET1e*-overexpressing plants introduces an intriguing aspect to this study. Aerial tuberization, a phenomenon not commonly observed in potatoes under normal growing conditions, is obviously linked to miR156 overexpression in *SlSWEET1e* overexpressors. SlSWEET1e overexpressors are reminiscent to miR156-overexpressing potato plants (Bhogale et al. 2014) and show increased levels of this miRNA, whereas mir172-overexpressing plants look different (Martin et al. 2009). The connection between *SlSWEET1e* overexpression and the induction of aerial tubers and miR156 raises questions about SWEET1 involvement in potential regulatory mechanisms influencing plant development via phloem-mobile miRNAs (Supplementary Figs. S16 and S17).

### Elucidation of the SWEET1e/g interactome

A high portion of StSWEET1g/SlSWEET1e-interacting proteins are cell wall-associated proteins (Table 1; Supplementary Table S3). The PMEI was identified in 6 yeast colonies independently and represents the second most abundant interaction partner of SWEET1e/g. SWEET1e/g also interacts with a cellulose synthase A subunit, a putative callose-binding protein, an unknown glycine-rich structural cell wall protein, a COBRA protein, and other cell wall proteins. In the context of COBRA, a GPI-anchored protein involved in cell wall development, its interaction with SWEET1e/g suggests a potential connection between sugar transport and cell wall dynamics.

Members of the COBRA family are involved in cell wall development across angiosperms (Brady et al. 2007). COBRA proteins belong to multigene families involved in cellulose synthesis and orientation, cell expansion, and cell wall biosynthesis. In Arabidopsis, the extracellular COBRA protein is a GPI-anchored protein that is involved in anisotropic expansion via cellulose microfibril orientation (Roudier et al. 2005). In tomato, overexpression of a SlCOBRA-like protein increased cell wall thickness in fruits, the number of collenchymatous cells, cellulose levels and pectin solubilization, as well as cell wall architecture in general (Cao et al. 2012). Mutation of COBRA genes often results in decreased cellulose content (Roudier et al. 2002). In maize plants, mutations of the COBRA gene *Brittle stalk2-like3* (Bk2L3) affects cellulose deposition, thereby affecting the ultrastructure of sieve element cell wall and carbon partitioning (Julius et al. 2021). The maize *Bk2L3* encodes a COBRA protein affecting cell wall formation, sucrose export from leaves, callose deposition, phloem cell wall ultrastructure, and thereby phloem pressure as well (Julius et al. 2021): *Bk2L3* mutants are defective in sucrose export from source leaves and accumulate high levels of soluble sugars and starch (Julius et al. 2021).

In Arabidopsis, the genetic suppressor of the *cobra* mutant phenotype MED16 accompanied with a reduced cellulose content could partially be rescued by increased PMEI. The MED16 locus regulates 2 PMEI genes, thereby affecting pectin methyl esterase activity and cell wall composition (Sorek et al. 2015). Also other StSWEET1g-interacting partners like WAT1 (*walls are thin* 1), protein 208, and arabinogalactan AGP16 are assumed to play a role in cell wall strengthening (Supplementary Fig. S15).

In this context, it is interesting to mention that in tomato plants, it was already described that the expression level of sucrose transporter genes correlates with the total area of the sieve element lumen. In a recent paper, the ultrastructural analysis of leaves of *SlSUT1* and *SlSUT2* antisense plants using electron microcopy revealed a significantly reduced total sieve element area that is correlated with reduced phloem sucrose exudation (Marco et al. 2021). There is obviously a link between sucrose transporter expression and sieve element cell wall biogenesis.

The main fraction of DEGs in *StSUT2*-RNAi plants published recently are also related to cell wall remodeling without affecting carbohydrate accumulation (Gong et al. 2023), among them several pectin esterase inhibitor proteins, suggesting an important role of SUT and SWEET proteins in cell wall remodeling and/or plasmodesmal transport.

It is obvious that a connection between cellulose biosynthesis, cell wall composition, sieve element cell wall thickness, and phloem turgor pressure exists affecting the efficiency of whole plant carbohydrate partitioning (Supplementary Fig. S16). StSWEET1g interaction partners could therefore affect the efficiency of phloem translocation.

### StSWEET1g/SlSWEET1e interactions at plasmodesmata

It is known from previous investigations that the subcellular localization of sucrose transporters is highly dynamic and depends on the oligomerization status, inhibitor treatments, high amounts of substrate (>500 mm sucrose), and the corresponding protein–protein interaction partner (Garg et al. 2020; Garg and Kühn 2022). It is therefore not surprising to see different subcellular localization patterns of heteromers in BiFC experiments compared to the monomeric forms (Fig. 8; Supplementary Fig. S11).

The most abundant interaction partner of StSWEET1g/SlSWEET1e is the LEAFY transcription factor (Table 1), a floral meristem identity gene essential for the formation of fertile flowers. In Arabidopsis, LEAFY targets many other floral meristem identity genes and floral homeotic genes (Yamaguchi 2021). In potato plants, the LEAFY homolog is involved in the transition to flowering and floral meristem identity. Knockouts led to indeterminate inflorescence development and replacement of flowers with leafy-like structures (Lebedeva et al. 2022).

It was shown that LEAFY is able to form homodimers and that homodimerization of the transcription factor is essential for the biological activity (Siriwardana and Lamb 2012). LEAFY is able to move through L1 to L3 layers of the shoot apex via plasmodesmata (Wu et al. 2003). Therefore, LEAFY-GFP is detectable in the nucleus and at plasmodesmata in Arabidopsis (Siriwardana and Lamb 2012). The partial localization of the SlSWEET1e-LEAFY complex in plasmodesmata-like structures may be functionally relevant (Supplementary Fig. S11).

The plasmodesmal localization of many heteromeric SlSWEET1e complexes (i.e. complexes with protein 208, LEAFY, and DIM1, Fig. 8; Supplementary Fig. S11) opens the question whether SWEET1e/g might also be involved in plasmodesmal functioning and permeability. First attempts to measure the level of callose in the transgenic plants revealed changes in callose content that correlate with the SWEET1e/g expression (and therefore also with the apoplasmic hexose content): more callose is detectable in leaves of *SlSWEET1e* overexpressors, whereas the callose content tends to be decreased in *StSWEET1g*-RNAi plants (Supplementary Fig. S18). This suggests a positive impact of StSWEET1g on callose accumulation, thereby potentially reducing plasmodesmal permeability. More work is needed to investigate whether changes in callose deposition are correlated with a change in plasmodesmal connectivity in these plants. It is also the question whether plasmodesmal permeability increases during development, which would be required e.g. in potato tubers during the switch from apoplasmic to symplasmic phloem unloading.

### Potential role in phloem unloading

It is obvious that a switch from apoplasmic to symplasmic phloem unloading requires intensive reconstruction of cell–cell connections and establishment of a sufficient number of plasmodesmata in order to allow symplasmic transport of sucrose out of the conducting phloem cells (Supplementary Fig. S17).

Several of the SWEET1g/e-interacting proteins are related to cell wall integrity or strength and stiffness: cellulose synthesis (cellulose synthase A, COBRA, and protein 208), pectin methylesterification (PMEI), or cell wall composition and structure (arabinogalactan, WAT1, and glycine-rich structural protein). It is known in potato that a switch occurs from apoplasmic to symplasmic phloem unloading between tuberization stage II, when the subapical region of the stolon starts swelling, and tuberization stage IV, when the diameter of the developing tuber is more than twice the diameter of the stolon (Supplementary Fig. S13; Viola et al. 2001). The sucrose efflux carrier StSWEET11 is discussed to be involved in this switch by limiting apoplasmic phloem unloading by blocking the leakage of sucrose into the apoplast (Abelenda et al. 2019). Not only SWEET carriers are assumed to be involved in apoplasmic phloem unloading but also StSUT1 seems to play a role herein (Kühn et al. 2003). StSWEET1g as well as StSUT1 expression decrease during tuber development, suggesting that they play in diminishing role in phloem unloading after the switch to symplasmic unloading (Supplementary Fig. S18). In parallel, the expression of StSWEET1g-interacting proteins involved in maintenance of cell wall integrity is also decreasing during tuber development (Supplementary Fig. S14). StSWEET1g and several of its interacting proteins are among the DEGs during tuber development (Shirani-Bidabadi et al. 2024). Downregulation of phloem unloading transporters involved in apoplasmic unloading, as well as downregulation of cell wall proteins involved in cell wall integrity (allowing disintegration of the cell wall), appears reasonable during a switch from apoplasmic to symplasmic phloem unloading (Supplementary Fig. S17).

## Conclusion

Although SWEET1e/g belongs to a large gene family and is most likely transporting hexoses, a clear impact on apoplasmic sugar ratio, expression level of *SP6A*, miR172 in source leaves, and thereby tuber production and flowering was demonstrated by manipulation of only one single gene. The cellular localization of StSWEET1g is still not known and it is still the question whether the direct interaction between StSWEET1g and StSUT1 is possible also in planta.

The interactome of this glucose carrier suggests a potential role in cell wall architecture, thereby affecting whole plant carbon partitioning, and potentially in plasmodesmata functioning. A more detailed investigation of the cell wall composition and architecture in these plants appears promising.

## Materials and methods

### Plant cultivation

#### Cultivating *S. tuberosum*

*S. tuberosum* L. var. Desiree plantlets were cultivated on 2-MS-media under sterile conditions. They were placed into a growth room with 22 °C, LD conditions (16 h of light, 8 h darkness). To keep a stock population of selected plants, stem cuttings were transferred into fresh medium every 4 to 6 wk. For experimental work, the rooted 4- to 6-wk-old cuttings were transplanted into type T soil and then placed into an air-conditioned (23 °C at day, 20 °C at night) phytochamber with constant LD conditions (16 h of light, 8 h of darkness). Pot size was either 12 × 12 × 12 or 15 × 15 × 20 cm. To prevent any infections, they were also inspected and treated with biological pesticides regularly. Phenotypes, flowering time, and tuberization were documented throughout the cultivation process. Leaf samples were taken from source leaves exposed to light. The time when samples were taken was kept consistent throughout the entire research process (11 Am to 1 Pm).

#### Cultivating *Nicotiana benthamiana*

Seeds were directly sawed onto type T soil. After germination, single, similarly sized seedlings were transferred into individual pots and grown in the greenhouse under LD conditions until they developed 4 to 6 leaves.

### Potato transformation

Potato transformation was performed as described earlier (Rocha-Sosa et al. 1989) using *Agrobacterium tumefaciens* strain pGV2260 (Deblaere et al. 1985). Potato leaf disks were cocultivated with agrobacteria for 4 wk in the dark. After 1 mo, first calli became visible and first sprouts appeared that were transferred onto root-inducing medium in the light. After 3 mo, small plants could be amplified and transferred to soil and be analyzed further. Five independent transformant lines were selected for each transformation. Different line numbers correspond to different transformation events and are derived from different calli.

### GATEWAY cloning

All inserts were cloned full length into the ENTRY vector pDONR207 using a 2-step procedure following the Invitrogen protocol for GATEWAY cloning using gene-specific att primers as given in Supplementary Table S1.

### Generation of the RNAi construct

The SWEET1e RNAi construct was generated by inserting an internal 100 bp *Spe*I–*Bam*HI fragment from the SlSWEET1e cDNA into the pUC-RNAi vector used before in previous experiments (Eggert et al. 2016) linearized sequentially by *Spe*I–*Bgl*II (BCUI). In a subsequent stem, the same fragment was ligated into the obtained vector linearized with *Bam*HI–*Xba*I, thereby inserting the fragment in opposite orientation positioned at the left and right side of a 200 bp intron of the GA20oxidase1 gene allowing stem–loop formation and intron splicing of the generated construct. The sequence identity between SlSWEET1e and StSWEET1g within the 100 bp *Spe*I–*Bam*HI fragment that was used for generation of the RNAi construct is 97.22% at the DNA level, the full-length cDNA sequences have 95.85% identity, and the identity at the amino acid level between the 2 proteins is 97%.

### Generation of SWEET1e overexpression construct

For SWEET1e overexpression, the SWEET1e cDNA was inserted into the binary vector pK7YWG2.0 (Karimi et al. 2005) with CaMV 35S promoter using GATEWAY technology. For subcellular localization, the potential interaction partners of SWEET1e were cloned in either pK7YWG2.0 or pH7RWG2.0 (Karimi et al. 2005) using GATEWAY technology.

### Isolating total RNA from plant tissue

RNA isolation was performed using TRIsure (Bioline) according to the manufacturer's protocol. Samples from 6-wk-old *S. tuberosum* plants were ground in liquid nitrogen using an electrical pestle (Heidolph) that was cooled using liquid N_2_. After homogenization, 1 mL of TRIsure reagent per 50 to 100 mg of ground sample was added. After mixing thoroughly, the samples were incubated at room temperature (RT) for 5 min. Secondly, 200 *μ*L of chloroform was added for every milliliter of TRIsure. Samples were shaken vigorously for 15 s until there were no layers visible. Following, they were incubated at RT for 2 to 3 min and centrifuged at 11,000 rpm for 10 min at 4 °C. The aqueous phase was then transferred into a new 1.5-mL tube. Five hundred microliters of isopropanol per milliliter of TRIsure was added. The tubes were inverted a few times. After incubation at RT for 10 min, the samples were centrifuged at 11,000 rpm for 10 min at 4 °C. The pellet was washed with 75% ethanol. Therefore, the test tubes were inverted a few times after adding ethanol and then centrifuged at 8,500 rpm for 5 min at 4 °C. Washing was repeated once with 1.5 mL of ethanol and 1 to 2 times with 1 mL. The pellet was air-dried for 10 to 15 min before being resuspended in 30 to 40 *μ*L of RNAse-free, autoclaved water. To help the pellet resuspend, the tubes were incubated in a thermal block at 65 °C for 10 min. Finally, the samples were centrifuged for 10 min at 11,000 rpm at RT. Extract concentrations were measured by NanoDrop, and RNA integrity was checked on a 1.2% agarose gel (0.5× TBE).

### Reverse transcription (cDNA synthesis)

Before synthesis of cDNA from a RNA template using reverse transcriptase, leftover genomic DNA had to be removed from the sample. For reverse transcription, 7.5 *μ*L of the sample containing 2,000 *μ*g RNA was transferred into PCR tubes. The desired RNA concentration was achieved by measuring it with NanoDrop. The PCR tube strips were then placed into a thermocycler preheated to 37 °C. After 30 min of incubation, 0.5 *μ*L EDTA was added to stop the reaction. To finish the DNA removal process, the samples were incubated for 10 more minutes at 65 °C.

### Real-time quantitative PCR

Real-time qPCR analysis was performed using reversely transcribed mRNA. The ChamQ Universal SYBR qPCR Master Mix was used. The SYBR Green dye included in this Master Mix bound to the amplified DNA strands enabling quantification via fluorescence measurements. The amount of PCR product was measured after every qPCR cycle. The cycles where the increase was exponential and above a given threshold were used for calculating the relative transcript amount via the 2^−ΔΔCt^ method. Real-time qPCR was performed using the C1000 Thermocycler and the CFX96 Real-Time System (BioRad). Each reaction mixture contained ddH_2_O, the cDNA template, ChamQ Universal SYBR qPCR Master Mix, as well as gene-specific forward and reverse primers as also listed in Supplementary Table S1. To ensure more accurate results for each sample, 2 technical replicates were pipetted and were then transferred into 96-well PCR plates and sealed using MicroAmp foils.

### Enzymatic sugar measurements

Determination of glucose, fructose, and sucrose contents in samples taken from *S. tuberosum* plants was performed as described previously (Stitt et al. 1989).

The measurements went as follows: approximately 50 mg fresh weight was prepared by incubating them in 0.5 mL 80% EtOH at 80 °C for 2 h until the leaf tissue lost its color. Then 20 *μ*L of the extract obtained was mixed with 780 *μ*L carbo buffer (including NAD, ATP, and glucose-6-P-dehydrogenase) in a cuvette. Extinction at 340 nm was measured as a base value before adding any additional enzymes. Next, the extinction was measured every time after each sequential addition of 2 *μ*L of each hexokinase, phosphoglucoisomerase, and invertase followed by 15 min of incubation at RT after mixing thoroughly.

### Enzymatic starch measurements

To measure starch contents in *S. tuberosum* leaf tissue, the decocted samples from previous sugar measurements were used. The pale leaf tissue was dried overnight at RT until the residual ethanol had evaporated. The dried samples then were weighed and finely ground before adding 400 *μ*L 0.2 m KOH. After mixing, the samples were incubated at 95 °C for 1 h to extract the starch.

Now the extract was neutralized using 80 *μ*L 1 m acetic acid. Afterward, the samples were centrifuged for 5 min at 10,000 × *g*. Fifty microliters of supernatant was mixed with 100 *μ*L 50 mm Na-acetate buffer (pH 5.0) containing 2 mg amyloglucosidase and incubated overnight at 55 °C. After cooling down, the process was very similar to the one described for sugar measurements.

### Infiltration and transient expression

Transient transformation of *N. benthamiana* was performed by infiltration with *A. tumefaciens* pGV2260 (Hellens et al. 2000) or *A. tumefaciens* EHA105 (Hellens et al. 2000).

Cells from the *A. tumefaciens* strain pGV2260 were used to induce a transient transformation in young *N. benthamiana* plants by performing so-called agroinfiltration. Previously transformed *A. tumefaciens* cells were plated on YEB medium containing the appropriate selective antibiotics. After 2 to 3 d of incubation at 29 °C, single colonies were used to inoculate 3 mL of liquid YEB medium. Those cultures were incubated at 29 °C and 250 rpm overnight. The next day, 1 to 2 mL of the cultures was used to start a 50 mL *A. tumefaciens* culture, to which 10 *μ*L acetosyringone (in DMSO) was added. Those cultures were incubated at 250 rpm overnight at 29 °C. The following day, the cells were harvested in 50-mL falcon tubes by 15 min of centrifugation at 3,500 rpm. The supernatant was carefully discarded, and the pellet was washed with ddH_2_O and centrifuged an additional time. Cells were diluted using infiltration buffer until an OD_600_ of approximately 0.5 was reached. These cell suspensions were incubated at RT for 2 to 3 h.

To infiltrate young *N. benthamiana* plants, the leaves first were punctured using a small needle. *A. tumefaciens* cells were introduced into the leaf tissue by placing a syringe (without a needle) on the abaxial side of the leaves. The described process was repeated until 3 to 4 leaves were fully saturated with the infiltrate. Expression of the desired genes was checked 3 to 5 d after infiltration with a confocal microscope (Zeiss LSM 800). GFP and YFP fluorescence was excited at 488 nm, RFP at 561 nm, and CFP at 405 nm. Negative controls were taken with exactly the same laser intensity and signal amplification.

### Yeast complementation

Yeast complementation assays were performed using the yeast strain EBY.SL1 (Mat a, ura 3-52, leu2-3, 112, his 3-Δ1, trp 1-289, mal2-8c, hxt1-18Δ::loxP, gal2Δ::loxP, suc2Δ::loxP, agt1Δ::loxP, mph2-3Δ::loxP, P suc2Δ::P HXT7loxP) (Krügel et al. 2013) or yeast strain EBY.VW4000 (MAT α leu2-3,112 ura3-52 trp1-289 his3-Δ 1 MAL2-8c SUC2 hxt17Δ hxt13Δ::loxP hxt15 Δ::loxP hxt16Δ::loxP hxt14Δ::loxP hxt12Δ::loxP hxt9Δ::loxP hxt11Δ::loxP hxt10Δ::loxP hxt8Δ::loxP hxt514Δ::loxP hxt2Δ::loxP hxt367Δ::loxP gal2Δ stl1Δ::loxP agt1Δ::loxP ydl247wΔ::loxP yjr160cΔ::loxP) (Wieczorke et al. 1999), which were transformed either with StSWEET1g, SlSWEET1e, SlSWEET12a, or StSUT1 cloned via GATEWAY technology in pDR196 GW or the empty vector pDR196 GW (kindly provided by Doris Rentsch). After selection on minimal medium without uracil, single colonies were grown on liquid minimal medium and adjusted to an OD_600 nm_ of 1 (corresponding to 3 × 10^7^ cells/mL). Equal amounts of cells were dropped on solid medium with 2% maltose, 2% sucrose, 2% glucose, or 2% maltose + the indicated amount of 2-DG. Cells were grown for 3 d at 30 °C.

### Split ubiquitin system

The split ubiquitin screen was conducted as previously described (Krügel et al. 2012). Large-scale yeast transformation was performed as described (www.umanitoba.ca/faculties/medicine/biochem/gietz/2HS.html). Yeast cells were transformed first with the bait construct. 3-AT is a competitive inhibitor of the *his3* gene product, and the specific 3-AT concentration in the selection medium needs to be determined to exclude autoactivation of the *his3* gene. Titration of the 3-AT concentration was performed by coexpression of the bait construct with the empty pNubGgate. The 3-AT concentration required to prevent autoactivation was determined to be 10 mm for SlSWEET1e.

Large-scale yeast transformation was performed according to the abovementioned protocol, cells were resuspended in 10 mL distilled water, and 500 *μ*L were plated on selection medium, respectively (20-cm-diameter Petri dishes) without trp, leu, his, and 10 mm 3-AT. In order to determine the transformation rate, 4 *μ*L were plated on selection medium without trp, leu (SD-LT), and the number of primary transformants was counted after 3 to 4 d.

### Apoplastic fluid by vacuum infiltration centrifugation

The isolation of the apoplasmic fluid from leaves was done by vacuum infiltration centrifugation basically as described by Abelenda et al. (2019). Collected source leaves were harvested, covered with aluminum foil, and placed on ice. The petioles and biggest veins of the leaves are carefully removed with a scalpel and the leaf is cut into approximately 1 cm × 1 cm squares. The leaf tissue is put into a large glass Petri dish and is first washed with ice-cold water and then submerged into cold infiltration buffer (90 mm HEPES and 90 mm MES, pH 5.5). Next, the Petri dish with the leaf tissue is put into a vacuum pump until the leaf tissue is infiltrated completely with infiltration buffer. This is indicated by a discoloration of the leaf. If necessary, the leaf tissue is stirred occasionally to make sure all pieces are completely submerged in the buffer. Once the infiltration is completed, the leaf tissue is washed with water and dried thoroughly. In order to extract the apoplastic fluid, approximately 5 g of plant material is placed onto a nylon mesh filter, which is folded into half, rolled up and placed, closed side down, into a 20-mL syringe with centered opening. The syringe is placed into a 50-mL falcon and centrifuged at 1,000 × *g* for 30 min, 4 °C. The nongreen liquid was considered as free of contaminants if glucose-6-phosphate dehydrogenase activity was absent. The apoplastic concentration of sucrose, fructose, and glucose was determined enzymatically as mentioned above.

### Esculin and 2-NDBG uptake in yeast cells

Esculin uptake experiments were basically performed as described by Gora et al. (2012) with small modifications. Yeast cells of the strain EBY.SL1 or EBY.VW4000 have been transformed with either the empty vector pDR196, StSUT1, StSWEET1g, SlSWEET1e, or SlSWEET12a in pDR196 and grown overnight for 16 h at 30 °C while shaking. Cells were collected for 5 min at 1,300 × *g* and incubated in 200 mL 1, 3, or 6 mm esculin (Sigma) in citrate buffer, adjusted at either pH 5 or pH 3 for 1 h at 30 °C with shaking. Cells were harvested again at 1,300 × *g* and washed with 200 mL of citrate buffer of the same pH. Cells were concentrated to an OD_600_ of approximately 1.0 and transferred to a black microtiter plate, and reading was performed with a Spectra Max M2 (Molecular Devices) with 367 nm excitation and fluorescence was detected at 454 nm emission wavelength. The relative fluorescence at OD_454_ was calculated with respect to the corresponding cell density (OD_600 nm_) that was measured in a transparent plate in parallel.

2-NDBG uptake experiments were basically performed after the identical protocol, but at a 2-NDBG concentration of 5.5 mm and in 25 mm phosphate buffer pH 7. Excitation was performed at 467 nm, and emission was detected at 538 nm. Relative arbitrary fluorescence units were calculated after background subtraction and with respect to the corresponding cell density.

### Callose determination

Callose determination was performed by a modification of the aniline blue fluorochrome method (Köhle et al. 1985). Three hundred milligrams of leaf material was cleared overnight (or 1 h) in EtOH at RT, dried on sterile filter paper, and homogenized in 1 N NaOH. Samples were heated to 80 °C for 30 min and centrifuged for 15 min at 10,000 rpm. Two hundred microliters of the supernatant corresponds to 20 mg FW.

Two hundred microliters of extract was mixed with 400 *μ*L of 0.1% (w/v) aniline blue WS in water (Merck, Darmstadt). After addition of 210 *μ*L of 1 N HCl, the color changes to deep blue, indicating neutral to acidic pH values. The final pH value was adjusted by addition of 590 *μ*L 1 m glycine/NaOH buffer (pH 9.5), and the tubes were mixed vigorously. During the following incubation for 30 min at 50 °C and further 30 min at RT, the aniline blues becomes almost completely decolorized. Fluorescence was measured using 200 *μ*L per sample in a dark microtiter plate with 405 nm excitation and 512 nm emission. Calibration curves were established using a freshly prepared solution of the 1,3-β-glucan in 1 N NaOH (1 mg/mL in 1 N NaOH in stepwise 1:5 dilutions).

### Phylogenetic analysis

Multiple sequence alignment was based on the amino acid sequences of all tomato SlSWEETs and potato StSWEETs listed in Supplementary Table S2. The alignment was generated using ClustalW (MegAlign Pro, DNAstar Lasergene 17), and the phylogenetic tree (Supplementary Fig. S3) was built by neighbor-joining BIONJ. The tree was rooted on midpoint branch.

### Statistical analysis

Statistical analysis was performed using Student's *t* test to determine the significance between 2 groups, and one-way ANOVA. Differences among the samples were considered significant at *P* < 0.05.

### Accession numbers

Sequence data from this article can be found in the GenBank/EMBL data libraries under accession numbers indicated in Supplementary Table S2.

## Supplementary Material

kiae602_Supplementary_Data

## Data Availability

The data underlying this article are available in the article and in its online supplementary material.

## Supplementary data

The following materials are available in the online version of this article.

**Supplementary Figure S1.** Coexpression analysis in tomato.

**Supplementary Figure S2.** Coexpression analysis in potato.

**Supplementary Figure S3.** Phylogenetic tree of 31 tomato SlSWEET genes and 35 potato StSWEET genes.

**Supplementary Figure S4.** Yeast complementation assay on different carbon sources.

**Supplementary Figure S5.** Cloning strategy of the RNAi construct used for generation of StSWEET1g-RNAi plants.

**Supplementary Figure S6.** Tuberization and plant size of transgenic potato plants grown under LD conditions.

**Supplementary Figure S7.** Flowering behavior of RNAi plants under LD conditions.

**Supplementary Figure S8.** Phenotype of transgenic lines grown under SD conditions in the phytochamber.

**Supplementary Figure S9.** Total sugar amount in transgenic potato plants and apoplasmic sugar ratio.

**Supplementary Figure S10.** Transcript quantification of plants grown under SD conditions via qPCR.

**Supplementary Figure S11.** Protein–protein interactions of *SlSWEET1e* in planta via BiFC.

**Supplementary Figure S12.** Negative control of BiFC experiments.

**Supplementary Figure S13.** Classification of tuber developmental stages.

**Supplementary Figure S14.** Gene expression of different StSWEET1g interaction partners and sugar transporters during tuber development.

**Supplementary Figure S15.** Summary of the effects of cell wall-residing proteins interacting with StSWEET1g.

**Supplementary Figure S16.** Summary of effects on apoplasmic sugar composition and expression of phloem-mobile miRNAs.

**Supplementary Figure S17.** Potential role of StSWEET1g during phloem loading and unloading.

**Supplementary Figure S18.** Callose quantification in leaves of transgenic potato plants.

**Supplementary Table S1.** List of primers used for RT-qPCR and GATEWAY cloning.

**Supplementary Table S2.** List of all potato and tomato SWEET genes with accessions.

**Supplementary Table S3.** List of StSWEET1g/SlSWEET1e-interacting candidates.

## Dive Curated Terms

The following phenotypic, genotypic, and functional terms are of significance to the work described in this paper:

AGP16 Gramene: AT2G46330
AGP16 Araport: AT2G46330

sucrose CHEBI: CHEBI:17992

ATP CHEBI: CHEBI:15422

MED16 Gramene: AT4G04920
MED16 Araport: AT4G04920

SUC2 Gramene: AT1G22710
SUC2 Araport: AT1G22710

SUT4 Gramene: XP_010695440.1
SUT4 Araport: XP_010695440.1

SWEET1 Gramene: At1g21460
SWEET1 Araport: At1g21460
