# Supplementary material for: The potato sugar transporter SWEET1g affects apoplasmic sugar ratio and phloem-mobile tuber- and flower-inducing signals
Source: Plant Physiol. 2024 Nov 7;197(1):kiae602. doi: 10.1093/plphys/kiae602 (PMC11663707; doi:10.1093/plphys/kiae602)
Supplement: kiae602_Supplementary_Data [file kiae602_supplementary_data.zip › PP2024RA02278DR1_Supplemental_Figures_1_18 1 2.pdf]

Supplementary Fig. S1

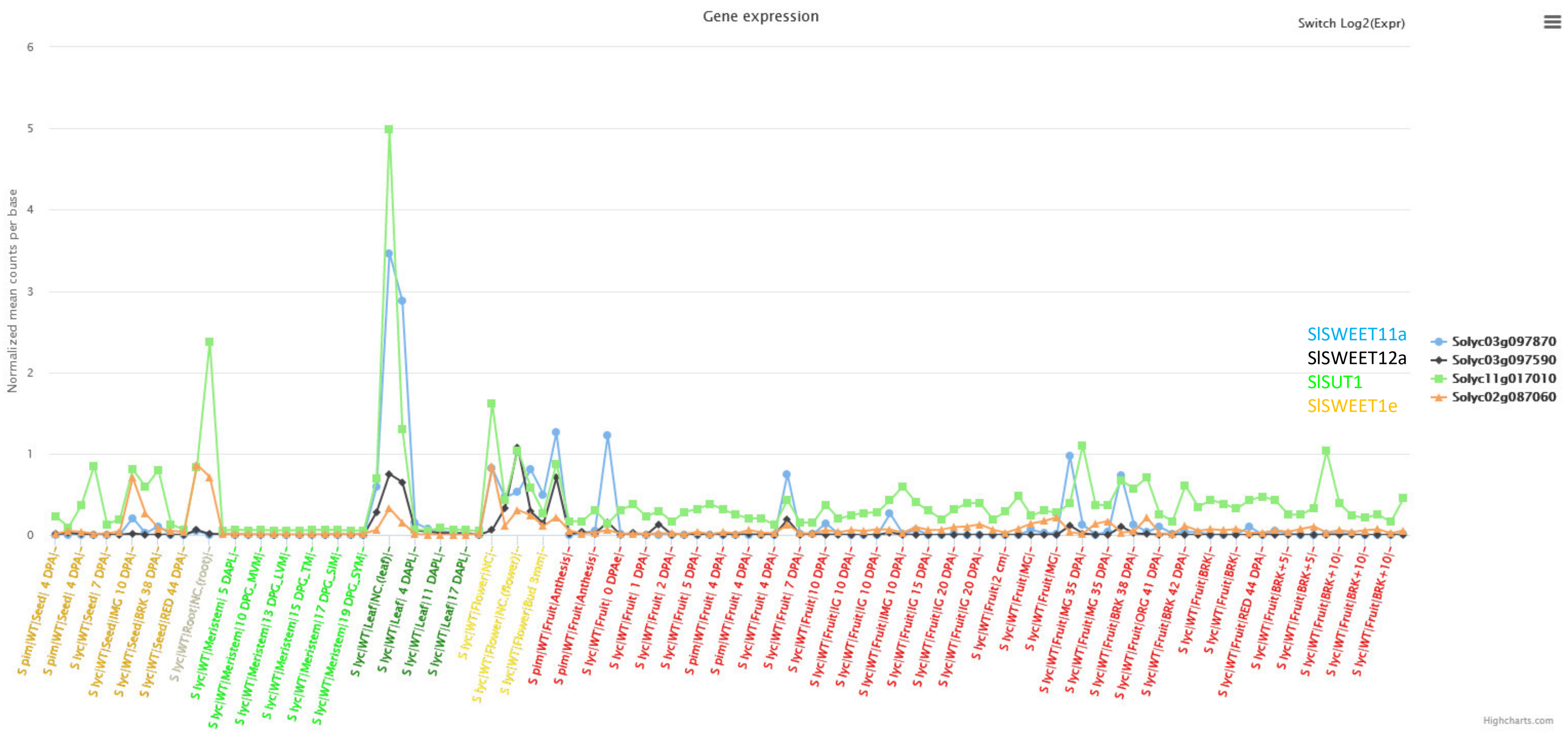

Suppl. Fig. S1. Co-expression analysis in tomato. Prediction of co-expression of SlSUT1 (Solyc11g017010), SlSWEET1e (Solyc06g60590), SlSWEET12a (Solyc03g097590) and the SlSWEET11a (Solyc03g097870) according to the TomExpress database (http://tomexpress.toulouse.inra.fr/aboutTomExpress). Co-expression analysis was performed on the 9th October 2023.

Supplementary Fig. S2

A StSWEET1g StSUT1

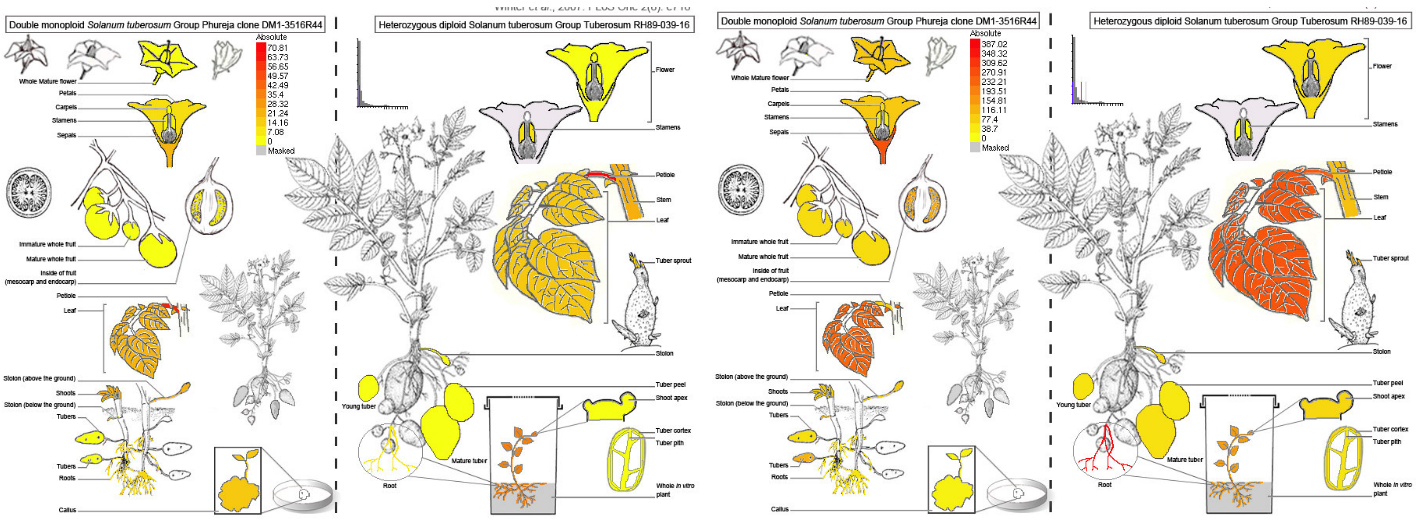

B

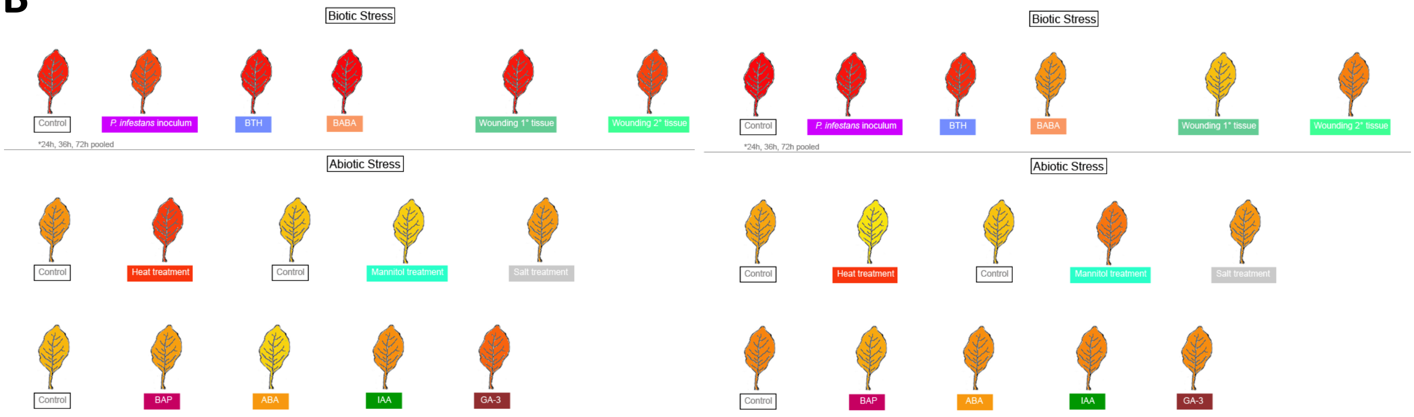

Fig. S2. Co-expression analysis in potato. Co-expression of *StSWEET1g* (PGSC0003DMG400013810) and *StSUT1* (PGSC0003DMG400009213) in potato plants during development (**A**) and in response to biotic and abiotic stresses (**B**) according to the potato eFP browser (https://bar.utoronto.ca/efp_potato/cgi-bin/efpWeb.cgi). Note that the expression of *StSUT1* is generally higher than the one of *StSWEET1g* (compare also with Fig. S14B and C).

Supplemental figure S3

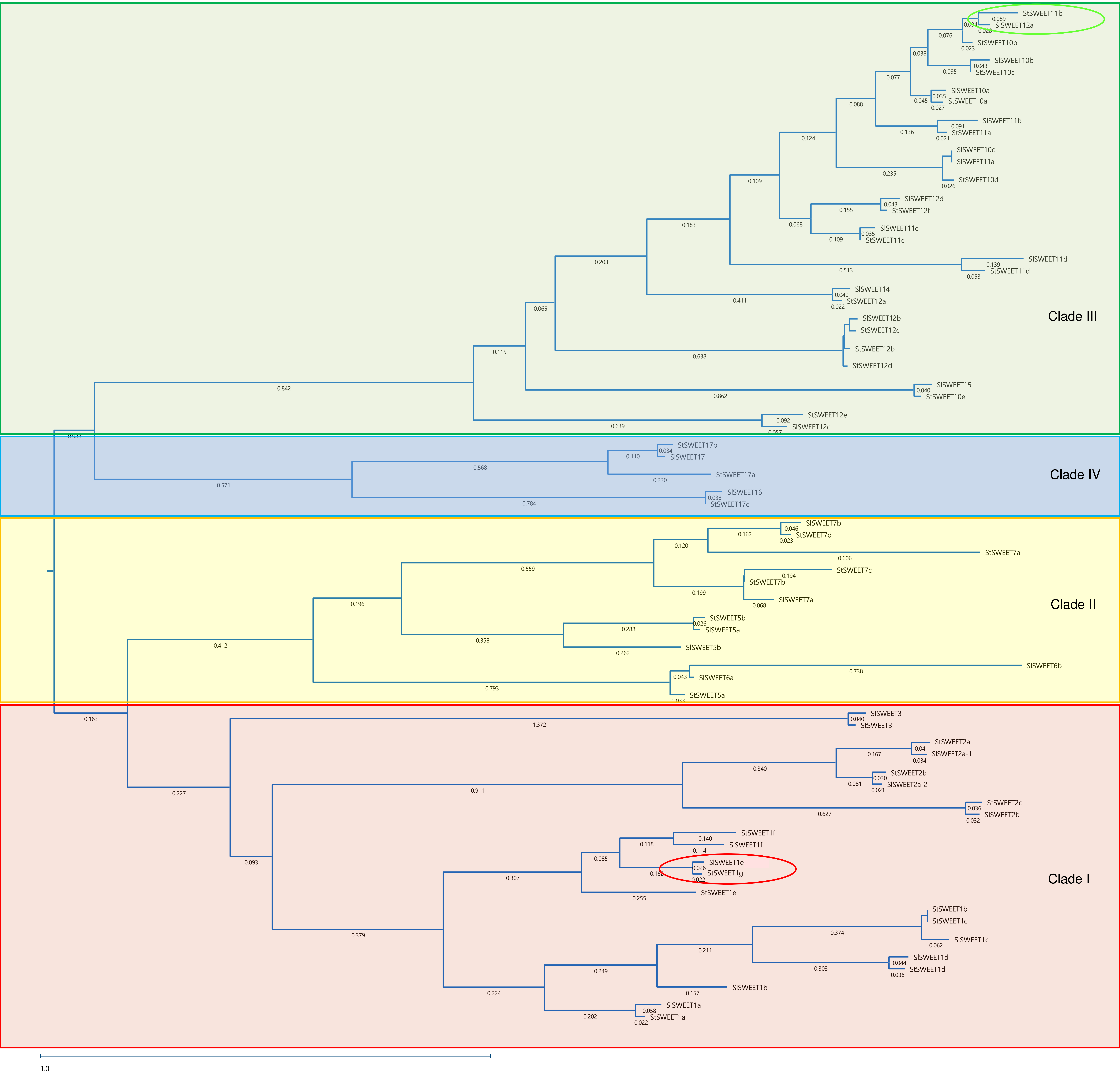

Fig. S3. Phylogenetic tree of 31 tomato SlSWEET genes and 35 potato StSWEET genes. Multiple sequence alignment based on amino acid sequences of 31 tomato SlSWEETs and 35 potato StSWEETs was generated using Clustal W (MegAlign Pro, DNAstar Lasergene 17) neighbor joining. The tree was rooted on midpoint branch. Scale bar indicates sequence distance. StSWEET1g and SlSWEET1e are highlighted in red.

Supplementary Figure S4

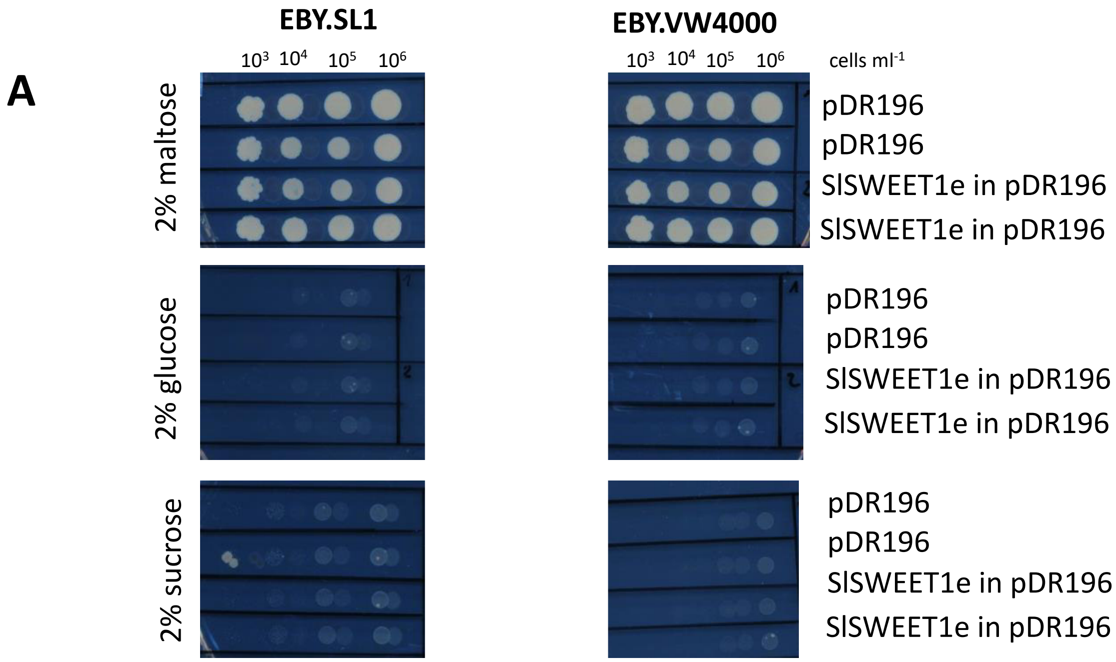

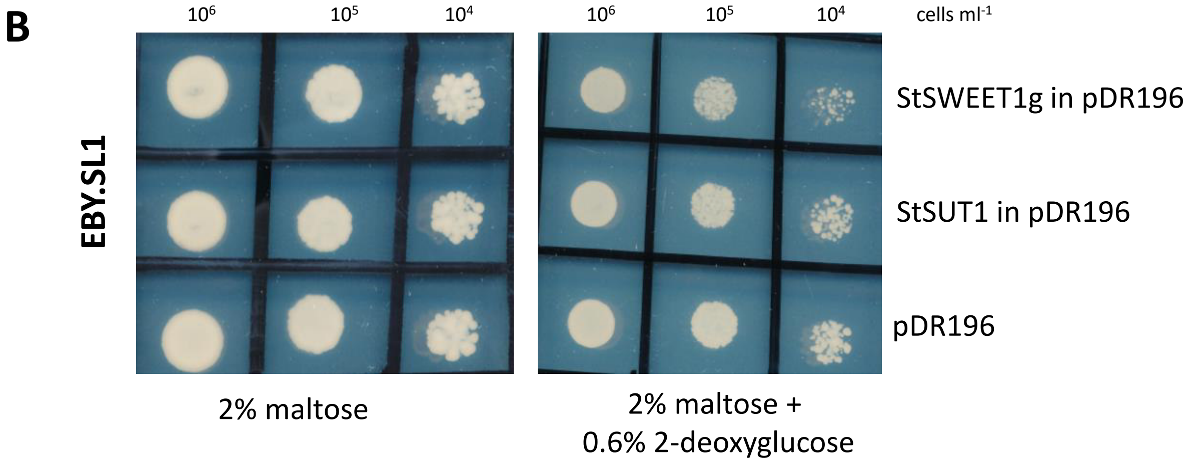

Fig. S4. Yeast complementation assay on different carbon sources. A. SlSWEET1e capacity to mediate yeast growth in different carbon sources was not successful. Glucose uptake defective yeast strains EBY.SL1 and EBY.VW4000 are able to grow on 2% maltose (upper row), but not on 2% glucose or 2% sucrose. Yeast strains transformed with *SlSWEET1e* in pDR196 neither. **B.** Yeast mutants EBY.SL1 transformed with the putative glucose transporter StSWEET1g show reduced growth in the presence of 0.6% of the toxic glucose analogue 2-deoxyglucose (right plate), whereas yeast cells transformed with *StSUT1* in pDR196 or the empty vector pDR196 cannot take up the toxic analogue.

Supplementary Figure S5A

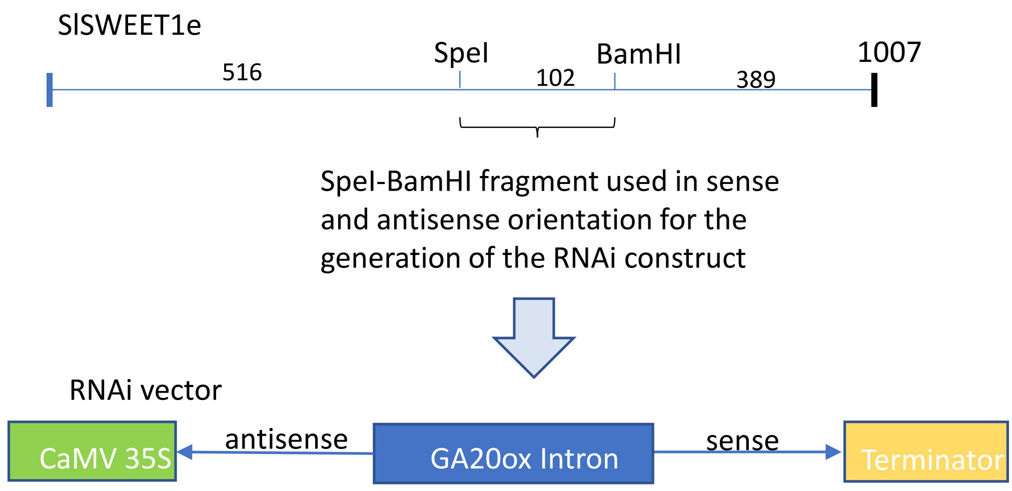

B

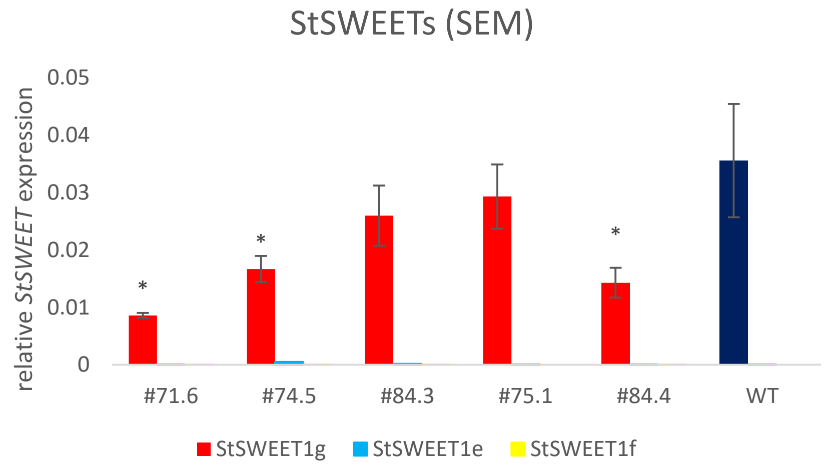

C

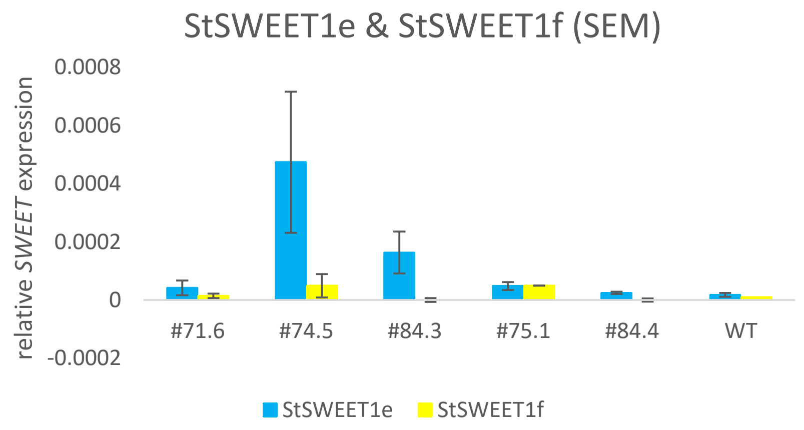

Fig. S5A. Cloning strategy of the RNAi construct used for generation of *StSWEET1g*-RNAi plants. B, C. Specificity test of *StSWEET1g* down-regulation. Real time qPCR with *StSWEET1e, StSWEET1g, and StSWEET1f*-specific primers revealed that only *StSWEET1g* expression is specifically down-regulated in *StSWEET1g*-RNAi plants. Note that expression of StSWEET1e and StSWEET1f expression is comparably low and therefore represented again in C. Three biological replicates were averaged and the TEF gene uses as a reference. Error bars indicate the standard error of the mean. T-test was performed with $p<0.05$ (*).

Supplementary Figure S6

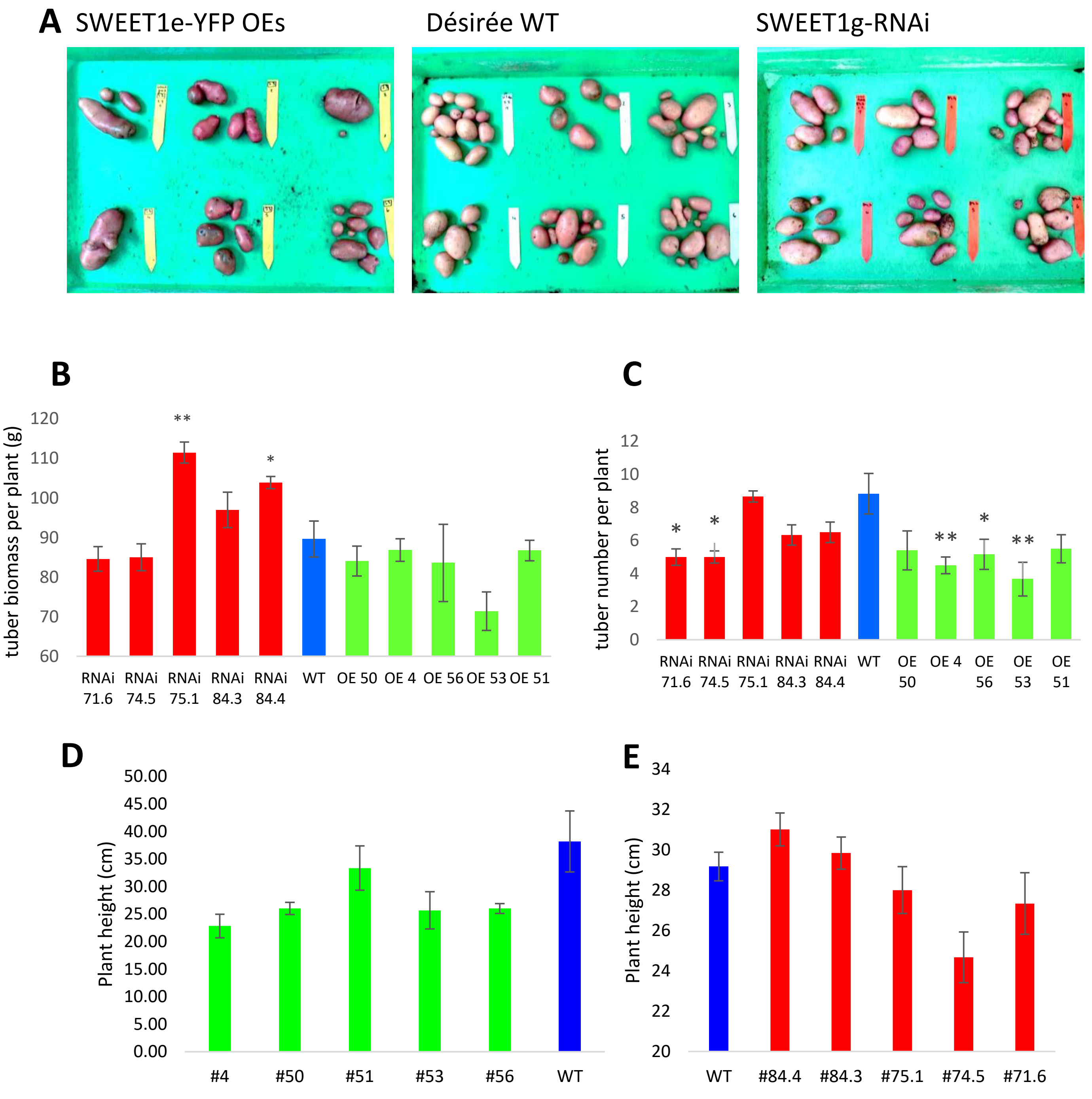

Fig. S6. Tuberization and plant size of transgenic potato plants grown under Long Day conditions. **A**. Tuber biomass of transgenic plants under Long Day conditions. **B.** Tuber biomass is increased in *SWEET1g*-RNAi plants (red) and reduced in *SWEET1e*-ovexpressing plants (green) under Long Days compared to the tuber biomass produced by wild type plants (blue). **C.** Tuber number is reduced in both sets of plants under Long Days. **D.** *SWEET1e*-overexpressors are smaller than wild type plants. **E.** Size of *SWEET1g*-RNAi plants is not significantly different from wild type plants. SEM is given. T-test was performed with $p<0.05$ (*), $p< 0.01$ (**).

Supplementary Figure S7

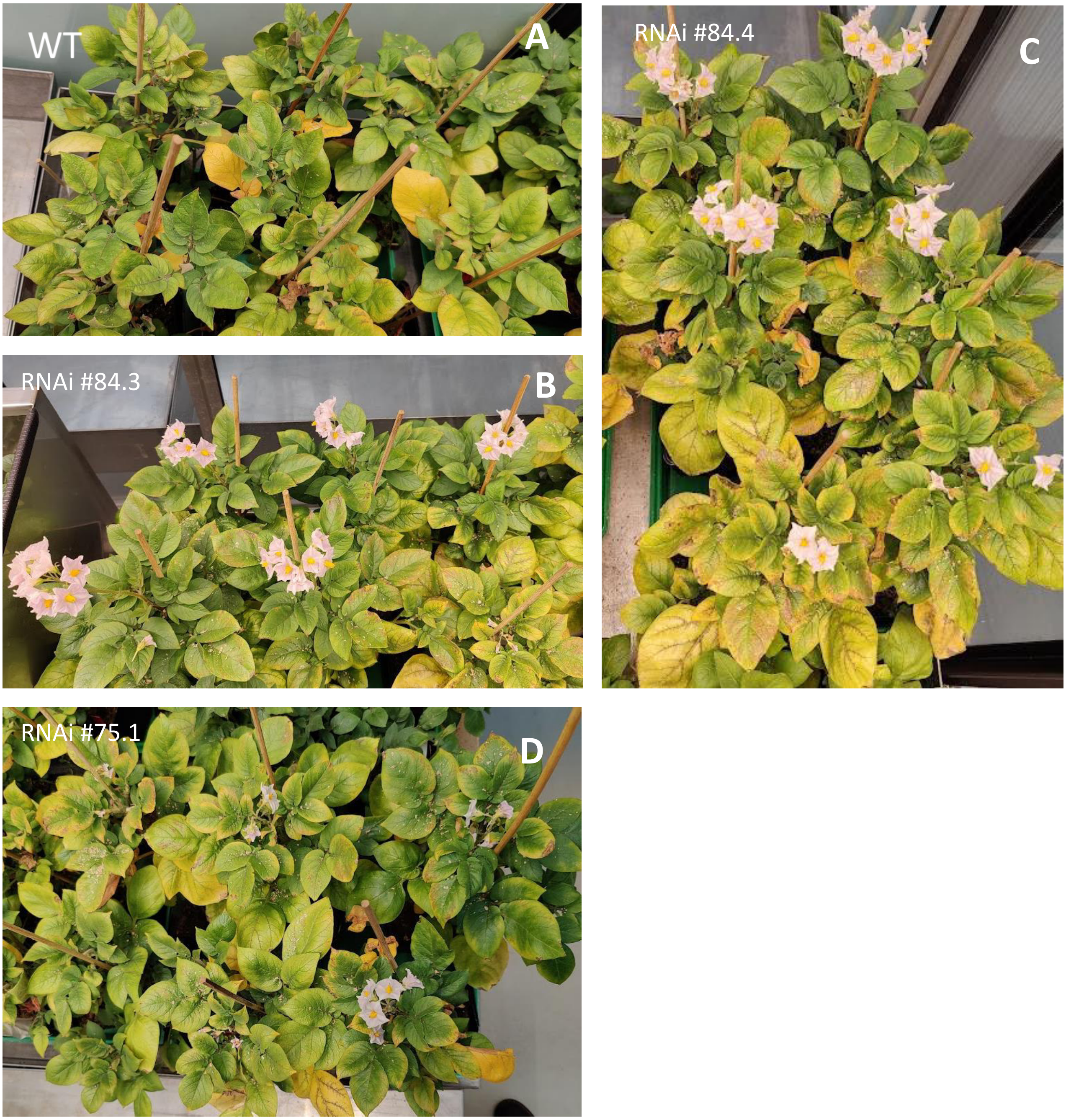

Fig. S7. **Flowering behavior of RNAi plants under Long day conditions**. *StSWEET1g*-RNAi plants are early flowering under Long Day conditions. **A.** Désirée wild type plants are not flowering after 5 weeks of growth under Long Day conditions**. B-D.** Three out of five transgenic *SWEET1g*-RNAi lines are early flowering under Long Day conditions (also shown in Fig. 3C).

Supplementary Figure S8

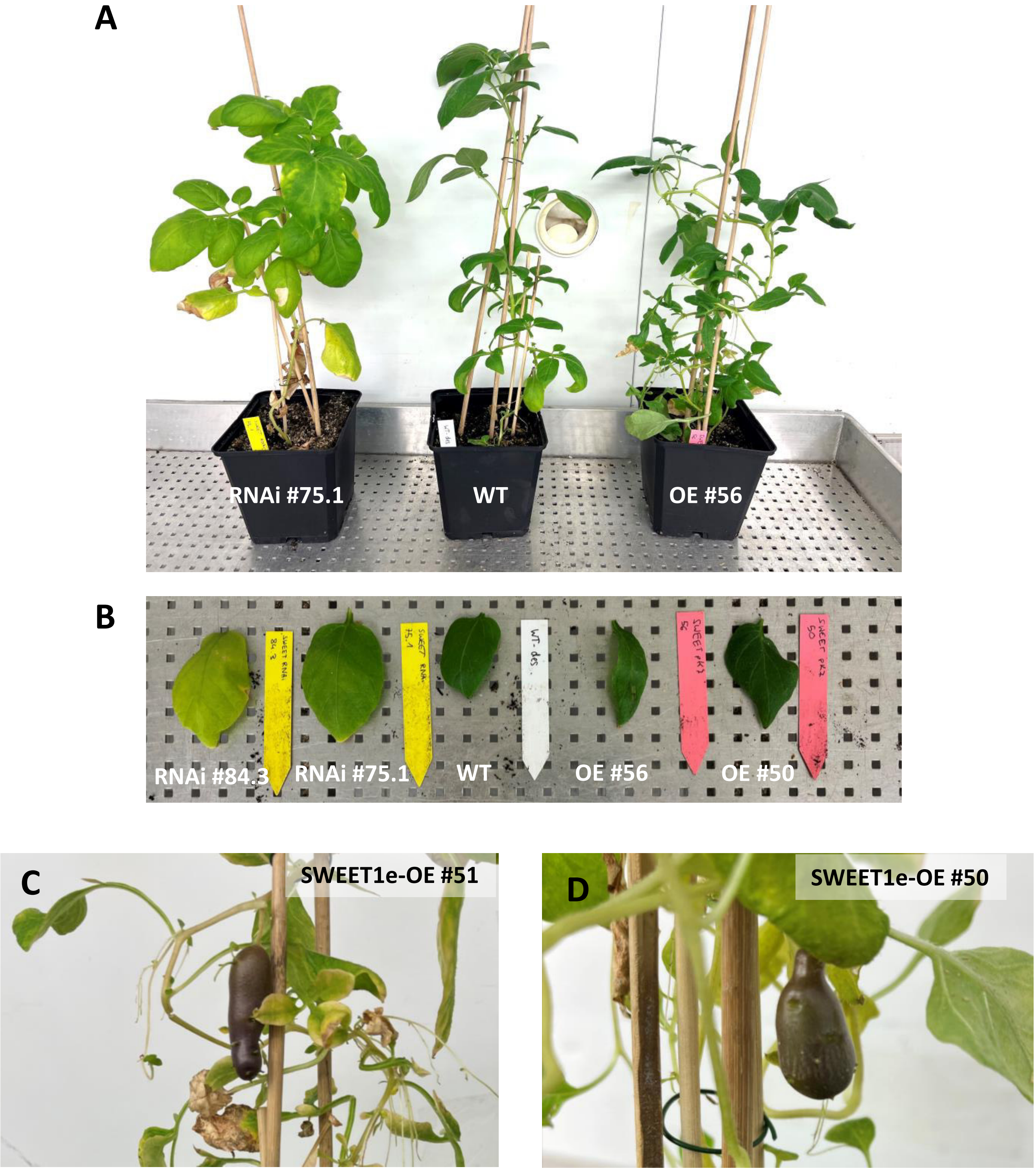

Fig. S8. Phenotype of transgenic lines grown under Short Day conditions in the phytochamber. A. Size of *StSWEET1g*-RNAi (left), WT (middle) and *SlSWEET1e*-overexpressing plants (right) under Short Day conditions. **B.** Leaf phenotype of *StSWEET1g*-RNAi (left), wild type (middle) and *SlSWEET1e*-overexpressing plants (right) under Short Day conditions. **C, D.** *SlSWEET1e*-overexpressors form aerial stolons and tubers under Short Day conditions, consistent with increased levels of miR156 (Eviatar-Ribak et al., 2013).

Supplementary Figure S9

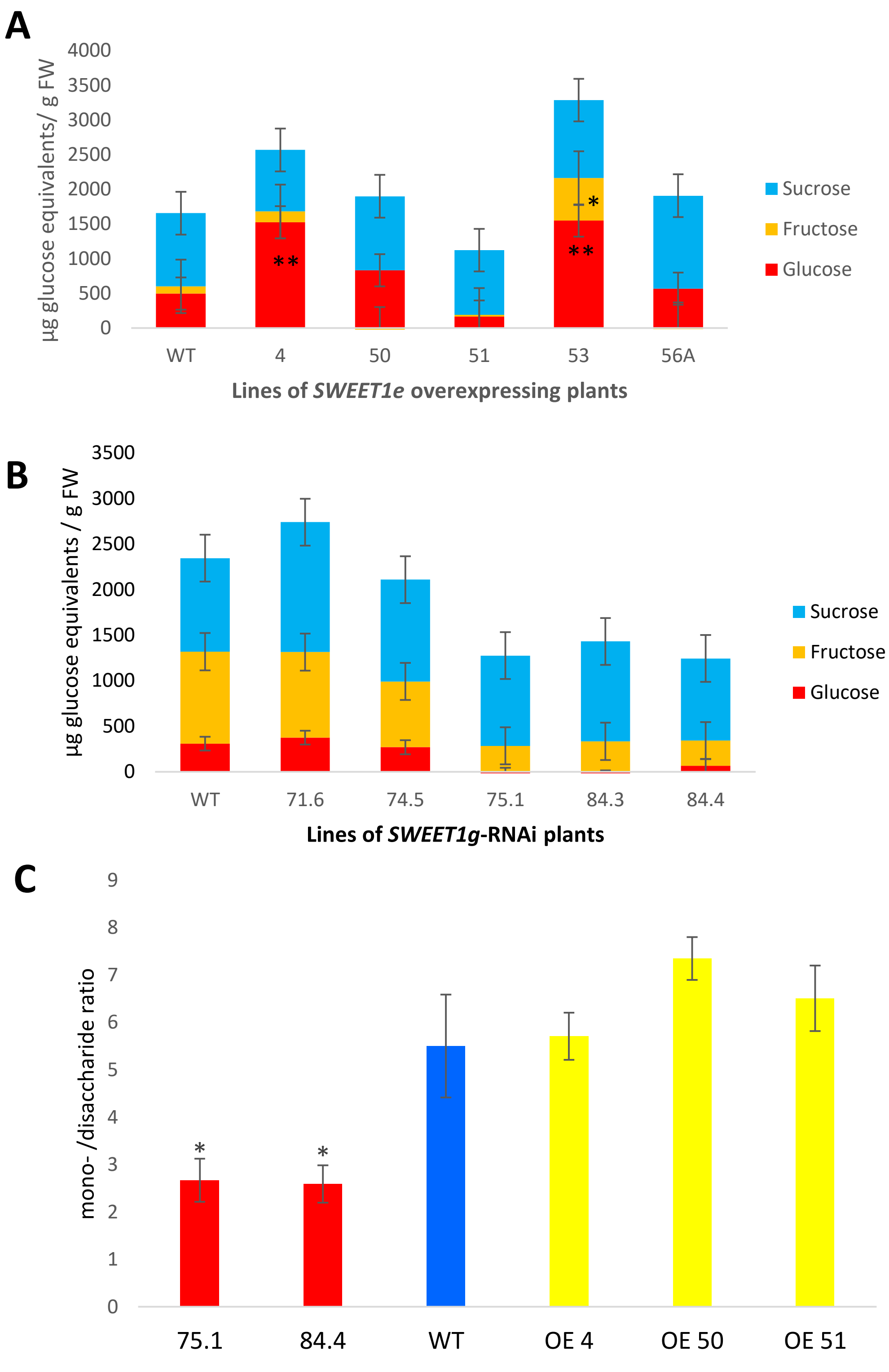

Fig. S9 Total sugar amount in transgenic potato plants and apoplasmic sugar ratio. A. Total amount of glucose, fructose and sucrose in source leaves of *SlSWEET1e*-overexpressing plants. **B.** Total amount of glucose, fructose and sucrose source leaves of *StSWEET1g*-RNAi plants under **Long day** conditions. Carbohydrates were determined enzymatically (Stitt et al. 1999). Note that mainly the glucose und fructose levels are affected in transgenic lines. **C.** Mono-/Disaccharide ratio of **apoplasmic** sugars of *StSWEET1g*-RNAi- (red), Désirée wild type (blue) and *SlSWEET1e*-overexpressing plants (yellow) grown under **Long day** conditions (number of samples between 6 and 28 replicates)**.** Ratio is similar to the ratio of **Short day**-grown plants shown in Fig. 4C. T-test with p<0.05 (*), p< 0.01 (**), p<0.001 (***).

Supplementary Figure S10: Short day conditions

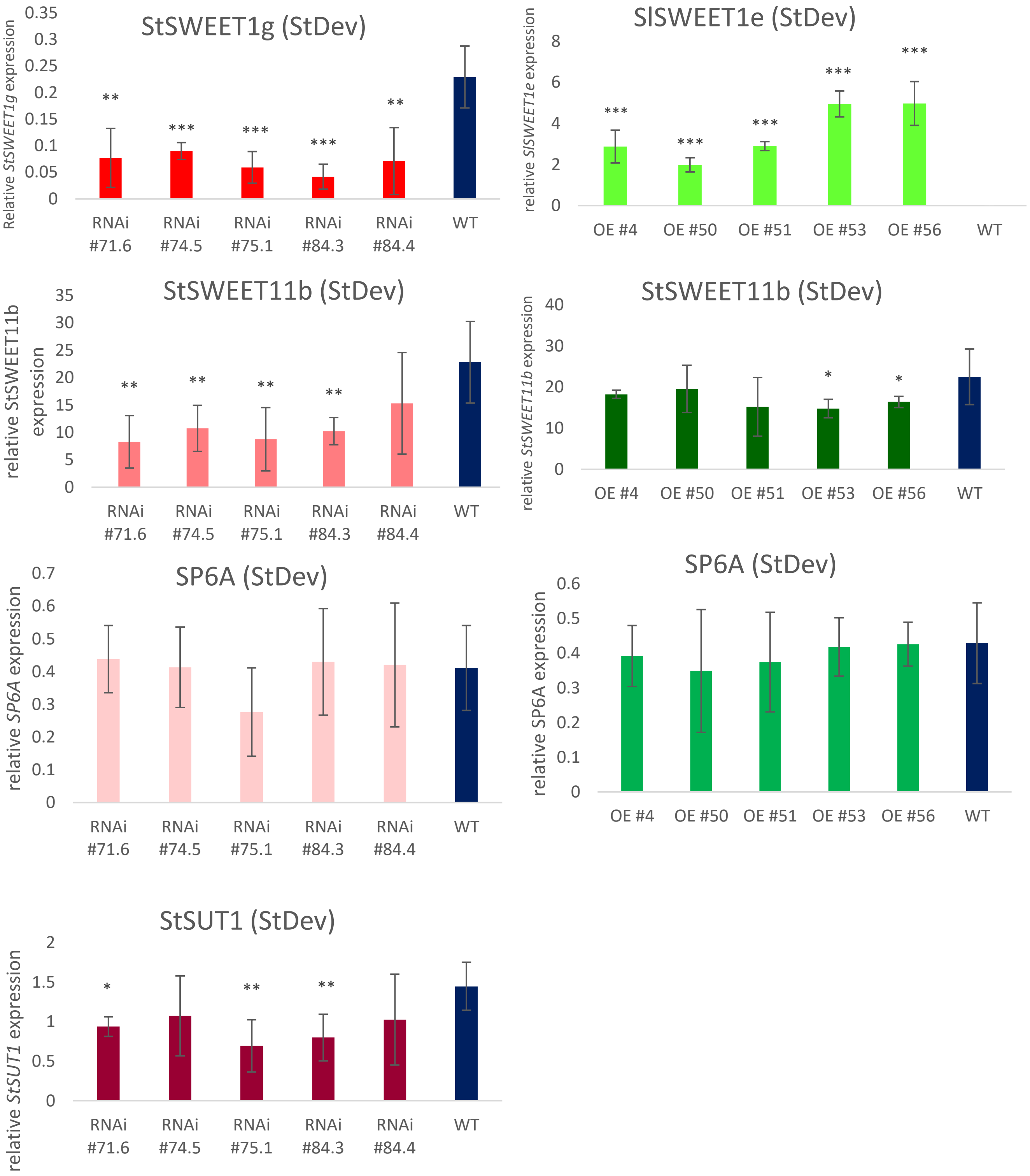

Fig. S10. **Transcript quantification via qPCR.** Real time qPCR analysis of the FT homologue *StSP6A, StSWEET11b* and *StSUT1* under **Short day** conditions in *SlSWEET1e*-overexpressing plants (**right side**) and *StSWEET1g*-RNAi plants (**left side**). Note that changes in *SP6A* expression are less pronounced than under **Long day** conditions (Fig. 5). *SlSWEET1e*-overexpressors produce reduced tuber biomass and aerial tubers under Short day conditions. Tuber yield of *StSWEET1g*-RNAi plants is not significantly increased under Short day conditions. The average of 6 replicates is shown. TEF was used as a reference gene, StDev is given. T-test with $p<0.05$ (*), $p< 0.01$ (**), $p<0.001$ (***).

Supplementary Fig. S11

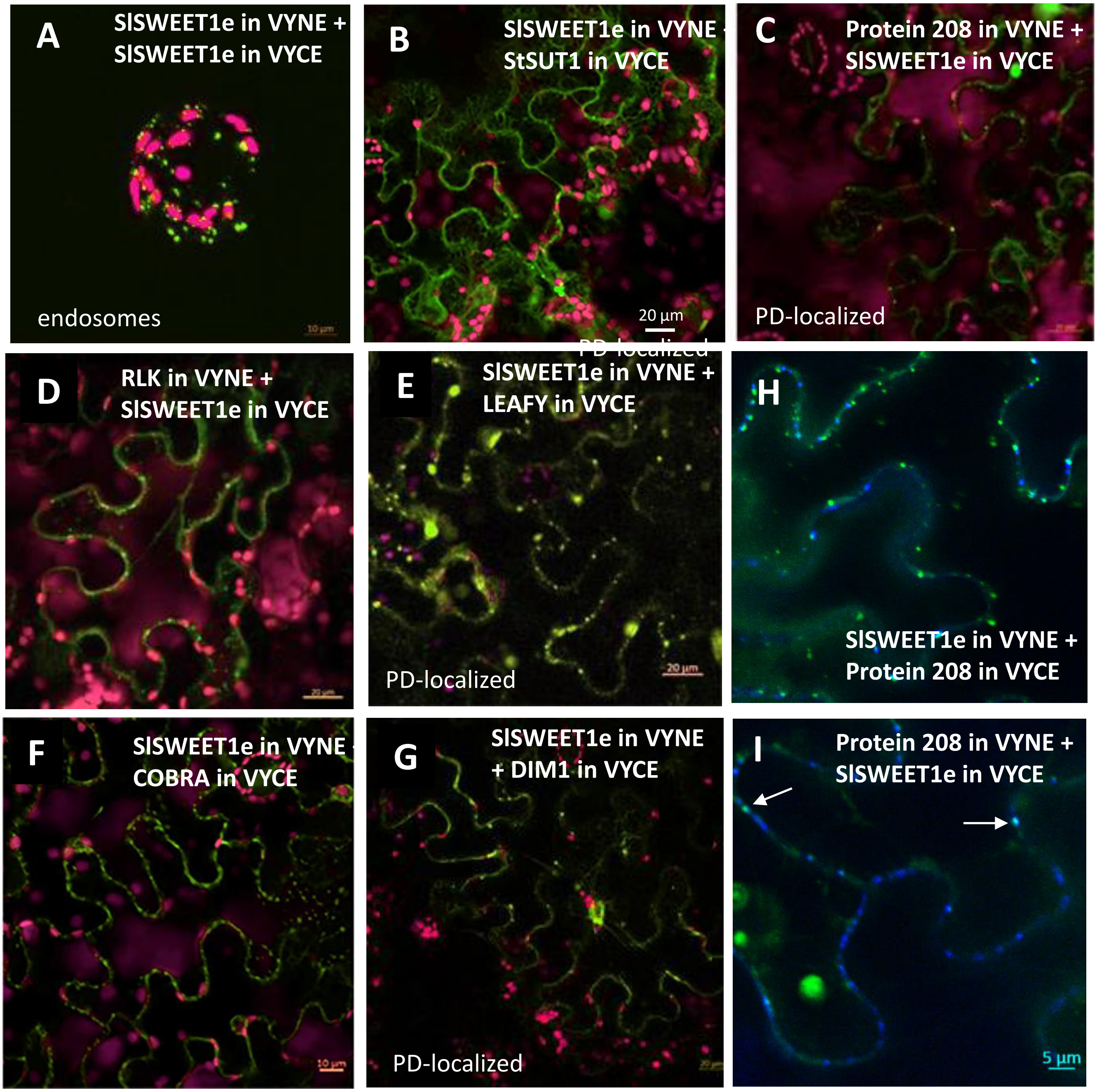

Fig. S11. Protein-protein interactions of *SlSWEET1e in planta* by bimolecular fluorescence complementation (BiFC). A. SlSWEET1e Homodimer formation induces internalization of the oligomeric complex in isolated protoplasts of *Nicotiana benthamiana* after infiltration of BiFC constructs. The homodimer is visible in endosomes. **B.** Heteromer formation between SlSWEET1e interacting with StSUT1 at the plasma membrane. **C.** Confirmation of interaction between the protein 208 and SlSWEET1e. **D.** Confirmation of interaction between RLK and SlSWEET1e. **E.** Confirmation of interaction between SlSWEET1 and LEAFY. **F.** Confirmation of interaction between SlSWEET1e and COBRA. **G.** Confirmation of interaction between SlSWEET1e and DIM1. Pictures were taken 3-4 d after infiltration. Note that interactions mainly take place in the cell periphery and that interaction changes subcellular localization of interaction partners (compared to Fig. 7). **H.** BiFC experiment with SlSWEET1e in VYNE and Protein 208 in VYCE. **I.** BiFC experiment with Protein 208 in VYNE and SlSWEET1e in VYCE. Pictures were taken 4 d after infiltration**.** Counterstaining of infiltrated leaves with aniline blue confirms co-localization of the heteromeric complexes between Protein 208 and SlSWEET1e with plasmodesmal callose (arrows). Aniline blue is shown in blue, YFP fluorescence from BiFC experiments is shown in green, co-localization is visible in whitish dots (arrows).

Supplementary Figure S12

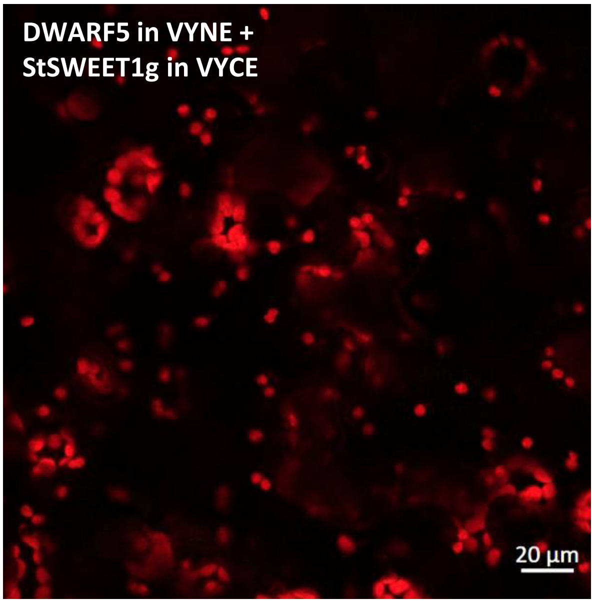

Fig. S12. Negative control of BiFC experiments. The sterol reductase DWF5 is not interacting with the glucose carrier StSWEET1g in BiFC Experiments, whereas the sterol reductase DIM1/DWF1 does (Fig. 8B). Image was taken under exactly the same settings as used for confocal analysis of BiFC experiments shown in Fig. 8 with the same laser intensity at 488 nm and the same voltage for signal amplifcation.

Supplementary Figure S13

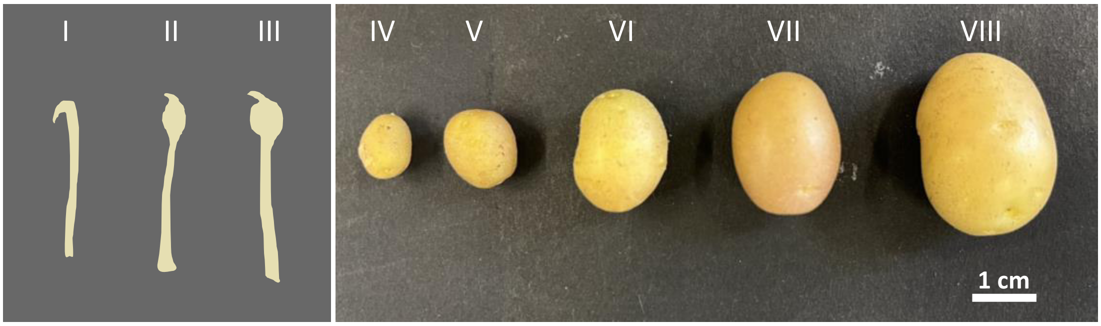

I	hooked stolon
II	slight swelling stolon below the apex
III	developing tuber is less than twice the diameter of the stolon
IV	developing tuber is more than twice the diameter of the stolon
V	0.6-1.5 g tuber
VI	1.5-2.5 g tuber
VII	2.5-5.0 g tuber
VIII	5.0-10.0 g tuber

Fig S13. Classification of tuber developmental stages. Tuber stages I-VIII from hooked stolons (stage I) to 5-10g tubers (stage VIII) were collected from phytochamber-grown potato plants after the switch to inductive short day conditions (8 h of light) and classified according to Kloosterman et al. (2005). Stages I-III are drawings and IV-VIII are photographs representative of each stage of tuber development.

Supplementary Figure S14A

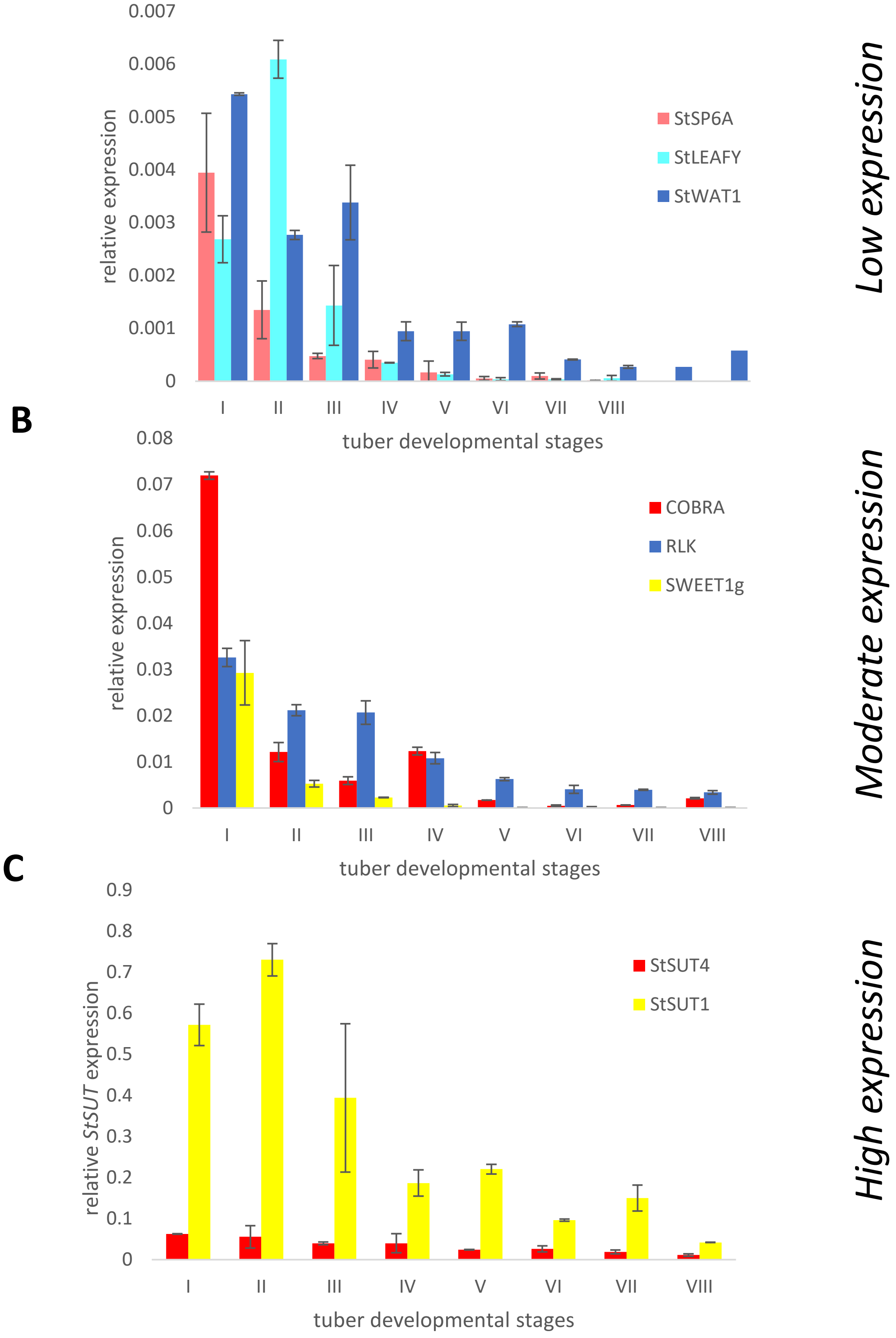

Fig S14. Gene expression of different StSWEET1g interaction partners and sugar transporters during tuber development. Tuber developmental were stages were classified according to Kloosterman et al. (2005) shown in Fig. S4. Four replicates are averaged for each measurement, TEF was used as a reference and the StDev is given. Note the difference in scale: genes are grouped according to their expression strength in A (low expression), B (moderate expression) or C. (high expression).

Supplementary Figure S15

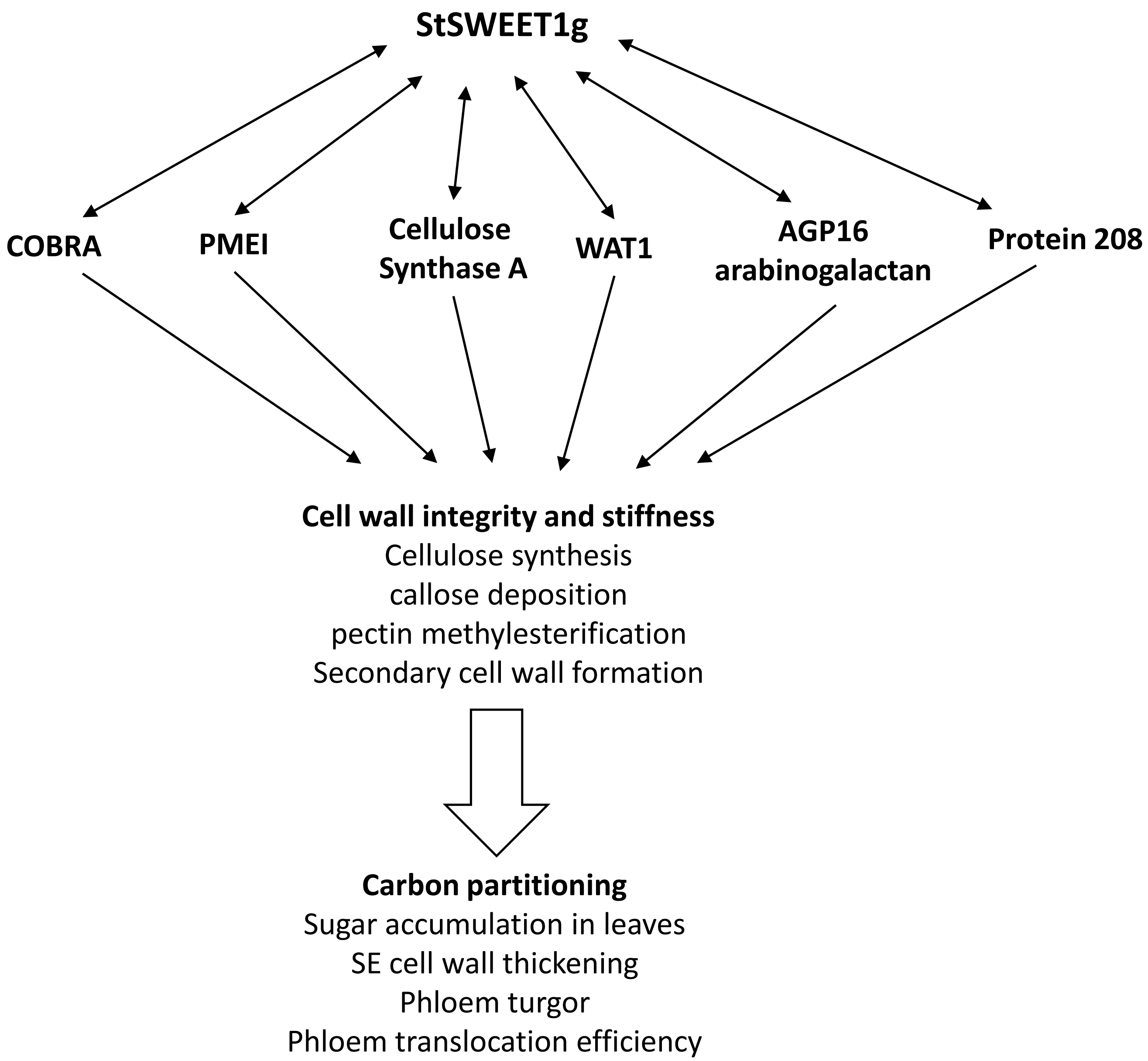

Fig. S15. **Summary of the effects of cell wall-residing proteins interacting with StSWEET1g**. 24% of the StSWEET1g-interacting proteins are cell wall proteins. Most of them are involved in the maintenance of cell wall integrity and strength or secondary cell wall formation. Down-regulation of StSWEET1g interacting proteins during tuber development might be correlated with cell wall disintegration during the switch from apoplasmic to symplasmic phloem unloading between developmental stage II and IV.

Supplementary Figure S16

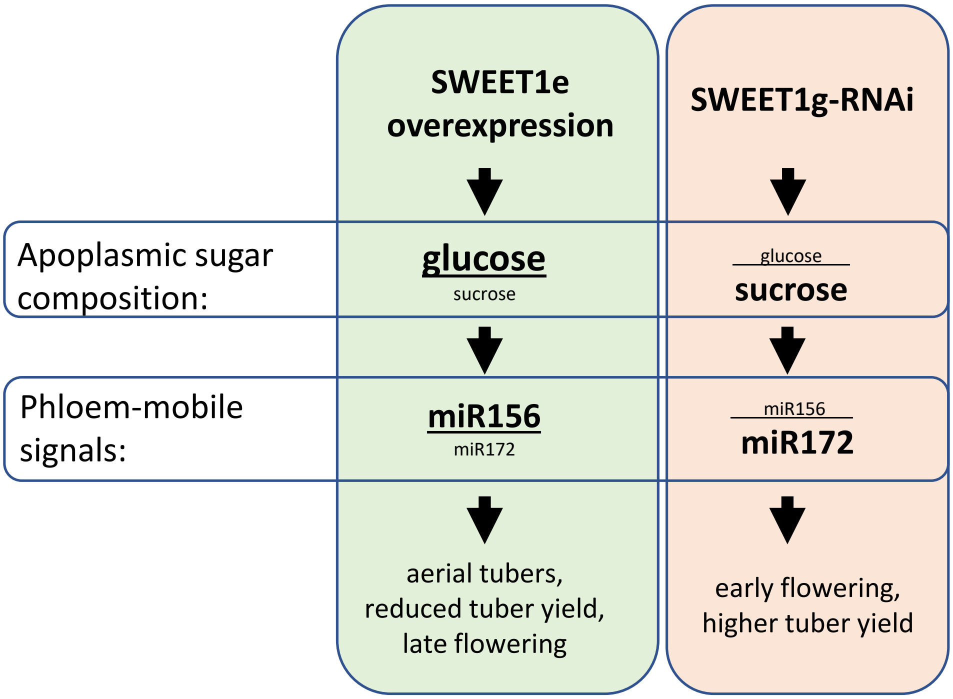

Fig. S16. Summary of effects on apoplasmic sugar composition and expression of phloem-mobile miRNAs. Altered SWEET1e/g expression mainly affects apoplasmic hexose content in SlSWEET1e-overexpressing and StSWEET1g-RNAi plants leading to a shift in the glucose/sucrose level. Whereas increased glucose/sucrose ratio in the apoplast leads to increased miR156 levels (Fig. 6A), a decreased glucose/sucrose ratio in the apoplast of StSWEET1g-RNAi results in increased miR172 levels under LD and SD conditions (Fig. 6A, B). Note that in parallel the general content of soluble sugars and callose in SlSWEET1e-overexpressing source leaves tend to increase, whereas total sugar content and callose levels decreased in StSWEET1g-RNAi plants (Fig. S8, Fig. S16).

Supplementary Figure S17

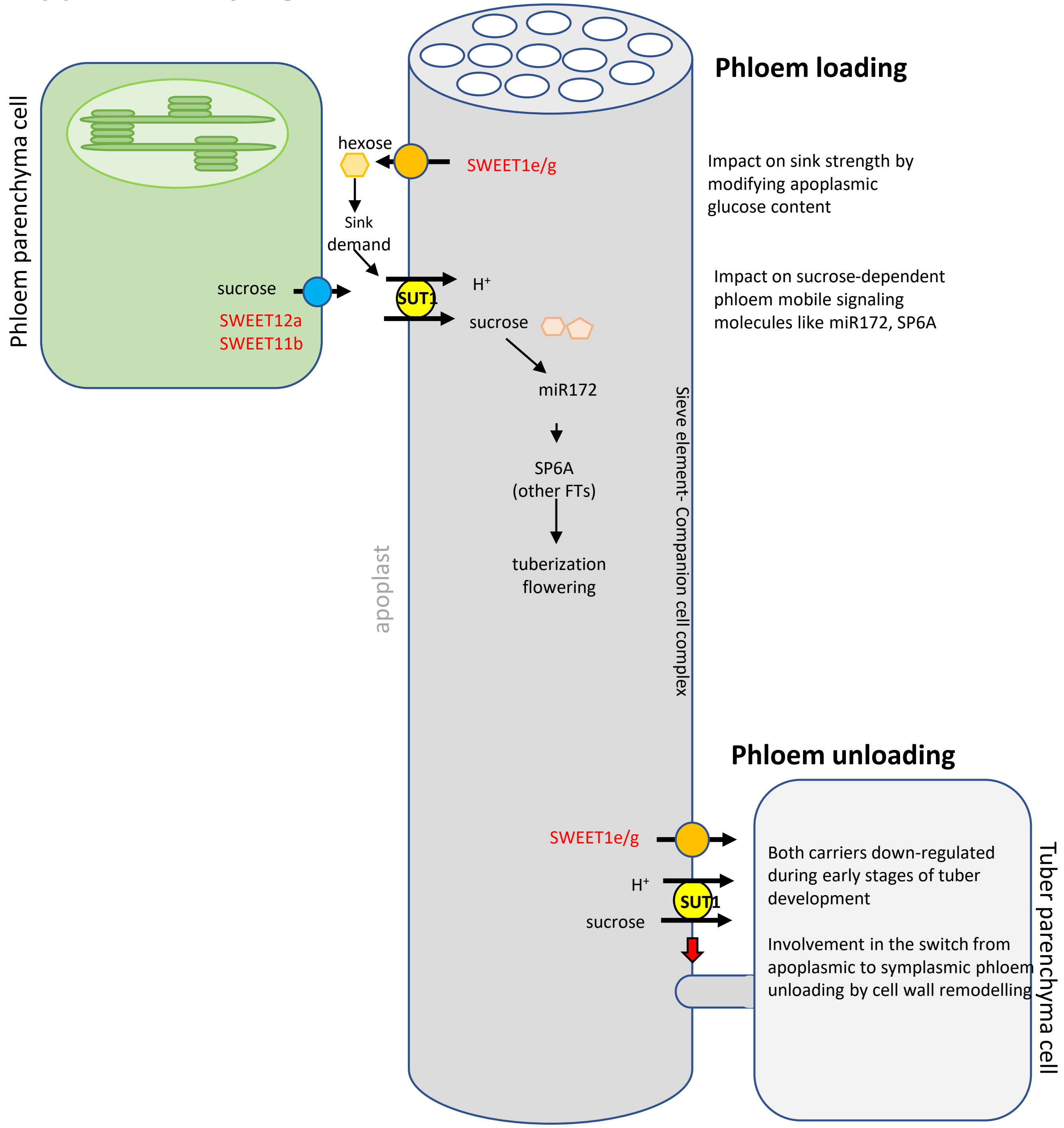

Fig. S17. Potential role of StSWEET1g during phloem loading and unloading. Hypothetical model illustrating the assumed function of StSWEET12 and StSWEET1g, which are tightly co-expressed with SUT1 according to expression databases. Functionality assays suggest a sucrose transport function for StSWEET12 and a glucose transport function for StSWEET1g (this work). It is suggested that StSWEET1g mediated efflux function triggers sink demand thereby affecting phloem loading via StSUT1 (whilst its cellular localization is still unknown). Phloem-mobile miR172 is sucrose-induced (Garg et al. 2021) and able (via AP2 transcription factors) to affect transcriptional efficiency of FT homologs (Coupland et al. 1995, Fornara et al. 2010) that are known to be involved in the onset of flowering and tuberization in potato (Navarro et al. 2011). **Potential role of StSWEET1g during phloem unloading**. Sucrose and glucose transporters of the SUT and SWEET family are down-regulated during early stages of tuber development (Supplementary figure S5) and active only during the early stages characterized by apoplasmic phloem unloading (Viola et al. 2009). Later, the switch to symplasmic phloem unloading is initiated via downregulation of cell wall stabilizing enzymes (like cellulose synthase A, PMEI, COBRA, arabinogalactan) that interact and are co-expressed with StSWEET1g.

Supplementary Figure S18

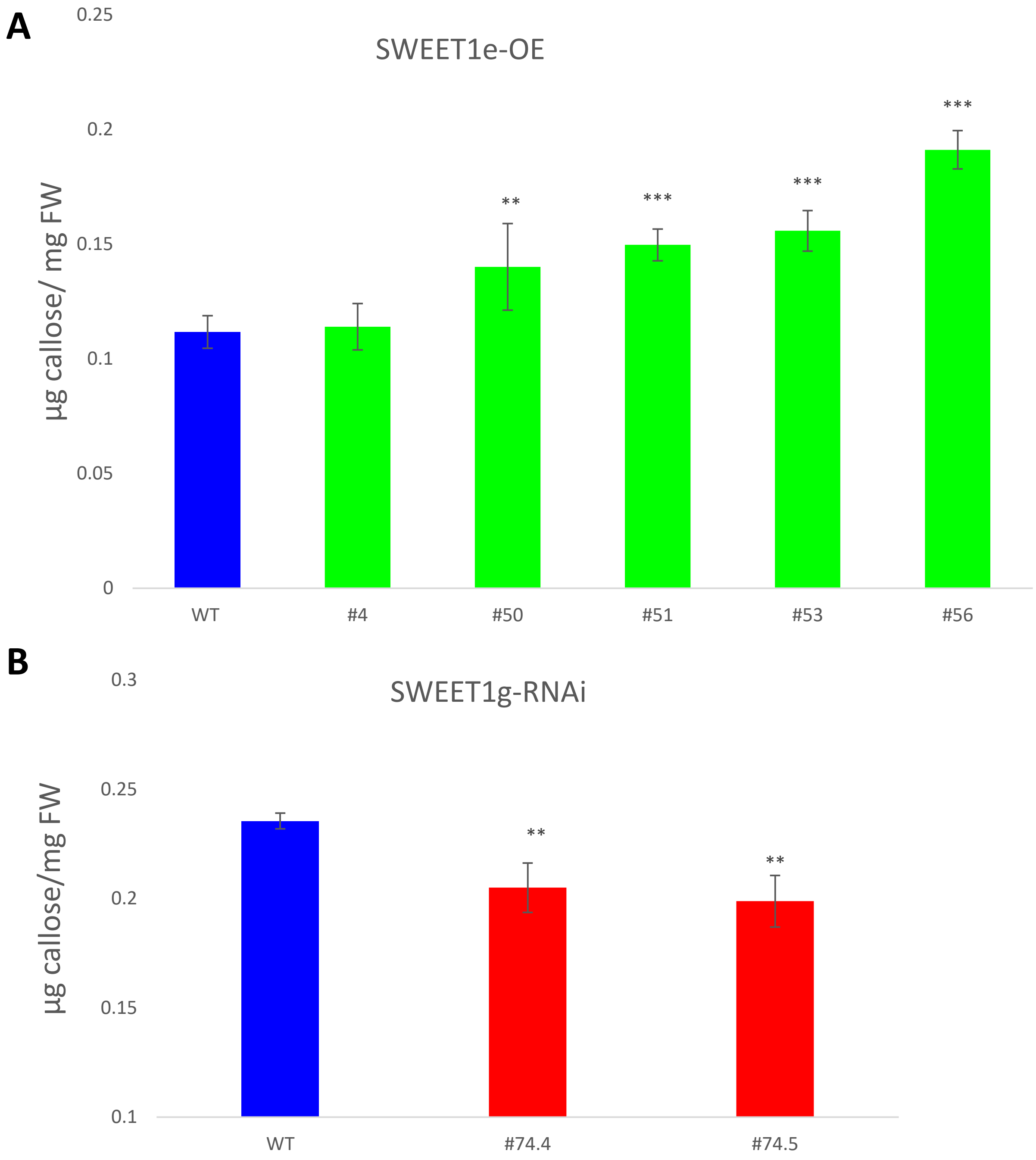

Fig. S18. Callose quantification in leaves of transgenic potato plants. Callose content in source leaves of *SlSWEET1e*-overexpressing **(A)** and *StSWEET1g*-RNAi plants **(B)** as determined by an aniline blue-based protocol according to Köhle et al. (1985). The average of 6 biological replicates is shown, the SEM is given. T-test with $p<0.05$ (*), $p< 0.01$ (**), $p<0.001$ (***).
