# Supplementary material for: The potato sugar transporter SWEET1g affects apoplasmic sugar ratio and phloem-mobile tuber- and flower-inducing signals
Source: Plant Physiol. 2024 Nov 7;197(1):kiae602. doi: 10.1093/plphys/kiae602 (PMC11663707; doi:10.1093/plphys/kiae602)
Supplement: kiae602_Supplementary_Data [file kiae602_supplementary_data.zip › PP2024RA02278DR1_Supplementary_Tables.pdf]

Supplementary Table S1: List of primers used for RT-qPCR and GATEWAY cloning.

Primer sequences for RT-qPCR:

**Gene name**	**fw**	**rev**
**StTEF**	AAC CAT GAC AAT GGC AGG AC	CCA AGT ATC GCA GCA AGT GG
**SlSWEET1e**	TAT CGC GGC GGT TAT TTC GG	AGC AGC CGT ACC ACA GAA GA
**StSWEET1g**	GCG GTT ATT TCA GTG CTT GC	GCG GTT ATT TCA GTG CTT GC
**StSUT1**	CAA TTT GGT TGG GCT CTT CA	AGT AGC CCG ACA ACT GGC TGA
**StSWEET11b**	GTGGTTGGTGGATTTGGTGC	TGCCTCACAATGCCTAAGGG
**SP5G**	GGT GTG TAG ACT TTG GTG TGG TTT	GGC CTC AAG GCA CAT CCA T
**SP6A**	GAC GAT CTT CGC AAC TTT TACA	CCT CAA GTT AGG GTC GCT TG
**StSWEET1e**	TGCTACCATCATCACCGTCG	TACCACGACAGCAAACGACA
**StSWEET1f**	GCTGCGGTTATCTCAGTGCT	TGGCATGTACTCCACGCTTT
**StLEAFY**	TGCTTCCAATGCTCTGAGGA	TCCATGCCCCAACATTCTCT
**StRLK**	GGCCTACGAAAACTTGGACC	TAGAAAGGGGAAGCCAGCAA
**StCOBRA**	TCCCCGAGTACCTGGAAAAC	CCATCACCAGCAGCAATGTT
**StProt208**	ATGGGGAGATGTTTGACGGT	GCCGGAGATGATAGAAGCCA
**miRNA156 RT primer**	GTC GTA TCC AGT GCA GGG TCC GAG GTA TTC GCA CTG GAT ACG ACG TGC TC	
**miRNA156 fw**	GCG GCG GTG ACA GAA GAG AGT	
**miR172 RT primer**	GTC GTA TCC AGT GCA GGG TCC GAG GTA TTC GCA CTG GAT ACG ACA TGC AG	
**miR172 fw**	CGG CGG TAG AAT CTT GAT GAT G	
**RT rev primer**		GTG CAG GGT CCG AGG T
**5SrRNA RT primer**	GTC GTA TCC AGT GCA GGG TCC GAG GTA TTC GCA CTG GAT ACG ACA GGG AT	
**5SrRNA fw**	GGA TGC GAT CAT ACC AGC ACT	

Primer sequences for GATEWAY cloning and sequencing:

**Gene name**	**fw**	**rev**
**StGWD**	AAA AAG CAG GCT TAA TGG ATT CTA TGC ATC TGT CAC ACT G	AGA AAG CTG GGT ACT GGG GTT GAG GTC GCG ATT G
**attB**	G GGG ACA AGT TTG TAC AAA AAA GCA GCG T	GGG GAC CAC TTT GTA CAA GAA AGC TGG GT
**pDONR207**	TCG CGT TAA CGC TAG CAT GGA TCT C	GTA ACA TCA GAG ATT TTG AGA CAC
**StLFY**	AAA AAG CAG GAT CCA TGG ACC CAG ATG CTT TCT CGG CG	AGA AAG CTG GGT AGA AAT GCA GCA GGT GAT CAG CAA C
**SlSWEEt12a**	AAA AAG CAG GCT TAA TGG TTT TTA ATC ACT GGG C	AGA AAG CTG GGT ATT AAG CCA CGG TTT GCA G
**Protein 208 with stop**	AA AAA GCA GGC TCA **ATG** GCG AAT CAA GGA GCA AAA	A GAA AGC TGG GTA **CTA** ACG AGC TCT TGT TTT GAC
**Protein 208 without stop**	AA AAA GCA GGC TCA **ATG** GCG AAT CAA GGA GCA AAA	A GAA AGC TGG GTA ACG AGC TCT TGT TTT GAC
**COBRA**	AAA AAG CAG GCT ACA TGG CAG TCA CCA CAT TTT TC	AGA AAG CTG GGT ATT GTC GCA CCA ACA TAA AAA CC
**RLK**	AAA AAG CAG GCT CAA TGG CGA CTC TCT CTT CCA CC	AGA AAG CTG GGT AGA GCT TAT TAG ATG ACA GTT GGT TG
**SlSWEET1e**	AAA AAD CAG GCT ATA TGG GTG GTC TTG TAC ATA CTA TAC	AGA AAG CTG GGT TCT AGA CTT GCT CAT GAA AAT TTG
**SlSWEET1g**	AAA AAG CAG GCT TAA TGG GTG GTC TTG TAC AAA CT	AGA AAG CTG GGT ACT AGA CTT GCT CAT GAA AAT TTG
**NPTII**	ACC GGA TCT GGA TCG TTT CG	TTG GTC CCT CAT TTC GAA CC
**StGA20ox 1st intron**	GGT ACG GAC CGT ACT ACT CTA	CCC CTA TAT AAT TTA AGT GGA AA

Supplementary Table S2: List of all potato and tomato SWEET genes with accessions.

Accession numbers

original accessions found by BLAST

potato	NCBI protein reference	NCBI gene locus	Spud DB
SWEET1a	XP_006356306	LOC102600613	Soltu.DM.04G025620.1
SWEET1b	XP_006356304.2	LOC102599952	Soltu.DM.04G025630.2
SWEET1c	XP_006356304.2	LOC102600282	Soltu.DM.04G025630.2
SWEET1d	XP_006356303	LOC102599625	Soltu.DM.04G025650.2
SWEET1e	XP_015160546	LOC102600141	Soltu.DM.06G017730.2
SWEET1f	XP_006366753	LOC102599475	Soltu.DM.06G017720.1
SWEET1g	XP_006366752	LOC102599149	Soltu.DM.06G017730.2
SWEET2a	XP_006355568	LOC102580097	Soltu.DM.02G014590.1
SWEET2b	XP_015168171	LOC102594034	Soltu.DM.03G000980.1
SWEET2c	XP_006348336	LOC102589550	Soltu.DM.07G023250.1
SWEET3	XP_015165601	LOC102586613	Soltu.DM.03G022840.1
SWEET5a	XP_006338549	LOC102587850	Soltu.DM.02G032820.1
SWEET5b	XP_006345190	LOC102583604	Soltu.DM.06G026530.1
SWEET7a	XP_015166307	LOC102603968	Soltu.DM.08G004840.1
SWEET7b	XP_006343813	LOC102600110	Soltu.DM.08G029710.1
SWEET7c	XP_006356389	LOC102601071	Soltu.DM.08G029710.1
SWEET7d	XP_006360745	LOC102590945	Soltu.DM.12G008510.1
SWEET10a	XP_015164326	LOC102580855	Soltu.DM.03G020090.1
SWEET10b	XP_006347633	LOC102600144	Soltu.DM.03G020080.2
SWEET10c	XP_006347634	LOC102600477	Soltu.DM.03G020070.1
SWEET10d	XP_006347635	LOC102600803	Soltu.DM.03G020060.1
SWEET10e	XP_006359431.2	LOC102582684	Soltu.DM.09G022920.1
SWEET11a	XP_015164325	LOC102580855	Soltu.DM.03G020100.1
SWEET11b	XP_006363981	LOC102598354	Soltu.DM.03G019570.1
SWEET11c	XP_006347346	LOC102587047	Soltu.DM.06G027950.1
SWEET11d	XP_006347482	LOC102603274	Soltu.DM.06G027970.1
SWEET12a	XP_006347632	LOC102599809	Soltu.DM.03G020110.1
SWEET12b	XP_015164323	LOC102581190	Soltu.DM.03G020050.1
SWEET12c	XP_015160613	LOC102603273	Soltu.DM.03G020030.1

SWEET12d	XP_015160615	LOC102603614	Soltu.DM.03G020020.1
SWEET12e	XP_015169738, XP_006368075	LOC102599419, LOC102591902	Soltu.DM.05G012430.2
SWEET12f	XP_006347347	LOC102587377	Soltu.DM.06G027960.1
SWEET17a	XP_006344690	LOC102584252	Soltu.DM.01G039600.1
SWEET17b	XP_006344691	LOC102584588	Soltu.DM.01G039600.1
SWEET17c	XP_006344692	LOC102584909	Soltu.DM.01G039610.1

**tomato**	**NCBI protein reference**	**NCBI gene locus**
SlNEC1	XP_004247459	LOC101259076
SlSWEET1a	XP_004237722	LOC101258946
SlSWEET1b	XP_004237723	LOC101259239
SlSWEET1c	XP_004237724	LOC101259534
SlSWEET1d	XP_010320027	LOC101244279
SlSWEET1e	XP_004242009	LOC101255412
SlSWEET1f	XP_004242288	LOC101248588
SlSWEET2a-1	XP_004233011	LOC101263567
SlSWEET2a-2		
SlSWEET2b	XP_010324406	LOC101249940
SlSWEET3	XP_010317476	LOC101252361
SlSWEET5a	XP_004236509	LOC101246817
SlSWEET5b	XP_004241538	LOC101266340
SlSWEET6a	XP_004233701	LOC101251355
SlSWEET7a	XP_004245483	LOC101246848
SlSWEET7b	XP_004252656	LOC101263002
SlSWEET10a		LOC101260802
SlSWEET10b	XP_004235333	LOC101263195
SlSWEET10c	XP_004235339	LOC101265012
SlSWEET11a	XP_004235326	LOC101261104
SlSWEET11b	XP_004235342	LOC101265905
SlSWEET11c	XP_004241452	LOC101265550
SlSWEET11d	XP_010322967	LOC101247336
SlSWEET12a	XP_004235334	LOC101263500
SlSWEET12b	XP_004235470	LOC101262399
SlSWEET12c	XP_004239304	LOC101255592

SlSWEET12d	XP_004242139	LOC101247627
SlSWEET14	XP_004235340	LOC101265318
SlSWEET16	XP_004230255	LOC101258901
SlSWEET17	XP_010315469	LOC101244429

Supplementary Table S3: List of StSWEET1g/SlSWEET1e-interacting candidates

No	Name	NCBI	Spud DB	Arabidopsis
1	LEAFY	NM_001288057	Soltu.DM.03G032420.1	AT5G61850
2	PMEI	XM_006362054.2	SoltuDM03G015630.1	AT5G62350
3	anti-muellerian hormone receptor type 2	XM_006366928.2	Soltu.DM.03G004250.1	AT3G50685
4	TIP4-1	CP055241.1 chromosomal, KAH0642479.1	Soltu.DM.08G015360.1	AT2G25810
5	StPDI1	NM_001288541.1	Soltu.DM.01G040080.1	AT2G47470
6	Glycin-rich cell wall protein	XM_006361128.2	Soltu.DM.06G018590.1	At5g46730.2
7	AAP1	NM_001318651.2	Soltu.DM.04G031760.1	AT5G49630
8	Selenoprotein T	XM_006366880.2	Soltu.DM.09G007630.1	AT3G47300
9	Selenoprotein K	XM_004250283.4	Soltu.DM.11G010960.1	AT4G08230
10	Receptor-like kinase	XM_006339552.2	Soltu.DM.01G033990.1	AT4G00300
11	Transmembrane protein 208	XM_006343517.2	Soltu.DM.07G001260.1	AT2G23940
12	COBRA protein	XM_006363519.2	Soltu.DM.01G020300.1	AT4G16120
13	Cellulose synthase A subunit	XM_006361662.2	Soltu.DM.08G011720.1	AT4G32410
14	G3BP-like protein	XM_015314363.1	Soltu.DM.09G017230.1	AT3G27150
15	Defensin	XM_015225605.2	Soltu.DM.11G013160.1	AT2G31957
16	PIP-type pTOM75	XM_006343909.1	Soltu.DM.08G027920.1	AT4G00430
17	WAT1-like (Umami transporter)	XM_006338475.2	Soltu.DM.02G032690.1	AT3G28050
18	Molybdate transporter	XM_006350438.2	Soltu.DM.03G021950.1	AT4G31940
19	TOM20	NM_001288206.1	Soltu.DM.07G025670.1	AT3G27080
20	Glukan water dikinase	XM_006358751.2	Soltu.DM.09G030970.1	AT5G26570
21	CORA protein	XM_015314115	Soltu.DM.09G030470.1	no similarities found
22	PS I subunit	XM_006361024.2	Soltu.DM.06G029590.1	AT1G08380

23	Tubulin beta-2 chain	XM_006350751.2	Soltu.DM.06G031950.1	AT5G23860
24	S-adenosyl-homocystein hydrolase	CP055242.1 chromosomal (KAH0637511)	Soltu.DM.09G029630.1	AT4G13940
25	plastid-lipid-associated protein	XM_006352030.2	Soltu.DM.11G016860.1	At3g26090
26	hypothetical protein	KAH0723560	Soltu.DM.10G027040.1	no similarities found
27	light-induced PS II	JX683432.1	Soltu.DM.07G028260.1	AT1G79040
28	E3 ubiquitin ligase	XM_006340096	Soltu.DM.11G022730.4	AT5G60580
29	TCP7 transcription factor	XM_015314355.1	Soltu.DM.04G005640.1	AT5G23280
30	StSUT1	NP_001305553.1	Soltu.DM.11G010180.1	AT1G22710
31	StDIM1	NM_001288265	Soltu.DM.02G003240.3	AT2G47470
